# Light–dark cycles may influence in situ soil bacterial networks and diurnally‐sensitive taxa

**DOI:** 10.1002/ece3.11018

**Published:** 2024-02-13

**Authors:** Nicole W. Fickling, Catherine A. Abbott, Joel E. Brame, Christian Cando‐Dumancela, Craig Liddicoat, Jake M. Robinson, Martin F. Breed

**Affiliations:** ^1^ College of Science and Engineering Flinders University Bedford Park South Australia Australia

**Keywords:** bacterial ecology, biodiversity, circadian rhythm, land cover, soil microbiome

## Abstract

Soil bacterial taxa have important functional roles in ecosystems (e.g. nutrient cycling, soil formation, plant health). Many factors influence their assembly and regulation, with land cover types (e.g. open woodlands, grasslands), land use types (e.g. nature reserves, urban green space) and plant–soil feedbacks being well‐studied factors. However, changes in soil bacterial communities in situ over light–dark cycles have received little attention, despite many plants and some bacteria having endogenous circadian rhythms that could influence soil bacterial communities. We sampled surface soils in situ across 24‐h light–dark cycles (at 00:00, 06:00, 12:00, 18:00) at two land cover types (remnant vegetation vs. cleared, grassy areas) and applied 16S rRNA amplicon sequencing to investigate changes in bacterial communities. We show that land cover type strongly affected soil bacterial diversity, with soils under native vegetation expressing 15.4%–16.4% lower alpha diversity but 4.9%–10.6% greater heterogeneity than soils under cleared vegetation. In addition, we report time‐dependent and site‐specific changes in bacterial network complexity and between 598–922 ASVs showing significant changes in relative abundance across times. Native site node degree (bacterial interactions) at the phylum level was 16.0% higher in the early morning than in the afternoon/evening. Our results demonstrate for the first time that light–dark cycles have subtle yet important effects on soil bacterial communities in situ and that land cover influences these dynamics. We provide a new view of soil microbial ecology and suggest that future studies should consider the time of day when sampling soil bacteria.

## Graphical abstract

This study explores the impact of light–dark cycles and land cover types on soil bacterial communities. It finds that land cover type significantly affects bacterial diversity and that there are time‐dependent variations in bacterial network complexity and relative abundance of individual taxa. These findings highlight the influence of light–dark cycles and land cover on soil bacterial composition, emphasising the importance of considering the time of day in soil bacteria sampling.

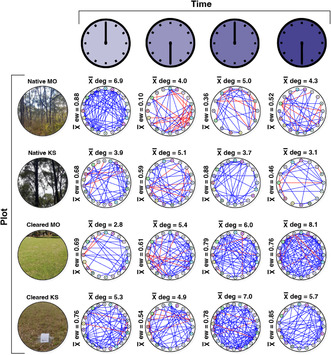

## 1 INTRODUCTION

Soil bacterial communities are highly diverse and are involved in many ecosystem processes (e.g. nutrient cycling, soil formation, plant health) (Liddicoat et al., 2023; van der Heijden et al., 2008). Our understanding of soil bacterial communities is increasing due in part to their links with human and ecosystem health (Liddicoat et al., 2019; Roslund et al., 2022; Singh et al., 2023) and advances in genomics (Berg et al., 2020; Liu et al., 2021; Mohr et al., 2022). While it is understood that plants and soil bacteria have a close relationship, further research is required to fully understand the breadth of connections and mechanisms involved. Filling this knowledge gap will contribute to an improved understanding of soil bacterial ecology, with potential implications for improving ecosystem integrity and soil microbial monitoring approaches.

Land cover types (e.g. open woodland, lawns) have strong effects on soil bacterial communities, as demonstrated in many studies where remnant vegetation has been compared to nearby land cover types such as lawn (Delgado‐Baquerizo et al., 2021; Hui et al., 2017; Liddicoat et al., 2019). While studies have reported higher soil bacterial heterogeneity across samples from remnant vegetation compared to cleared or urban lawns (Delgado‐Baquerizo et al., 2021) and the occurrence of notable bacterial functional groups in soils (e.g. butyrate‐producers) (Liddicoat et al., 2020, 2023; Roslund et al., 2020), further research is needed to improve knowledge on how land cover type and factors such as light–dark cycles interact to influence soil bacterial communities.

Plants display diurnal cycles due to endogenous circadian clock genes and *zeitgebers*—external cues that regulate organismal processes to a circadian rhythm or light–dark cycle (from the German terms Zeit ‘time’ + geber ‘giver’; Hörnlein & Bolhuis, 2021). While many circadian clock genes function independently of light, zeitgebers such as light or temperature are crucial for maintaining light–dark cycles (Hörnlein & Bolhuis, 2021; Staley et al., 2017). While some bacteria, such as cyanobacteria, display true circadian rhythms via circadian clock genes, many bacterial taxa do not possess these genes (Kondo et al., 1993). Despite this, all bacteria may still be subject to the influence of zeitgebers such as light, temperature, plant processes via plant–soil feedbacks and bacteria–bacteria interactions (Hörnlein & Bolhuis, 2021; Kelly et al., 2019; van der Meer et al., 2007). Plants and soils, for example, are intertwined primarily through the *rhizosphere*—the interface between roots and soil (Haichar et al., 2008). In often commensal relationships, plants make exudates available to bacteria (Badri & Vivanco, 2009; Haichar et al., 2008), while bacteria contribute to plants via increased access to soil nutrients and reduced presence of pathogenic bacteria, and these processes can fluctuate over short timeframes (Canarini et al., 2019; Doornbos et al., 2012). Therefore, while not all soil bacteria exhibit *true* circadian rhythms, soil bacterial communities may undergo light–dark cycles in their community composition due to wider ecological processes and zeitgebers.

Light–dark cycles of soil bacterial communities have been studied in greenhouse experiments with model and agricultural plant species *Arabidopsis thaliana* (Lu et al., 2021; Staley et al., 2017), rice (Zhao, Ma, et al., 2021) and barley (Baraniya et al., 2018). These studies have generally indicated that plants deposit carbon and other exudates into the soil (rhizodeposition) over light–dark cycles, generally to a greater degree during daylight hours, which affects both soil bacterial population growth and community composition. Additionally, the bacterial gene expression profiles in rhizospheres can vary across light–dark cycles. Staley et al. (2017), for example, detected cycling peaks of soil rhizosphere bacterial glycolysis genes at the opposite phase to the *A. thaliana* plants. Despite this, we found only one study on the effect of light–dark cycles on soil bacterial community composition outside of these model plant systems (Landesman et al., 2019). Landesman et al. (2019) assessed soil bacterial community changes over diurnal, sub‐seasonal (6‐weeks) and seasonal timescales. While they did not report soil bacterial community changes across the light–dark cycle, their analyses focussed on relatively coarse‐level alpha and beta diversity of sampled soil bacterial communities. Many studies have analysed the activity or growth of soil bacteria over light–dark cycles and short temporal scales but did not characterise soil bacterial communities. For example, Monard et al. (2021) measured volatile organic compounds emitted by soil microbial communities and detected higher fluxes during light hours, while Lünsmann et al. (2016) highlighted the varied expression of genes related to the metabolism of organic root exudates across day and night. There is also a substantial body of literature addressing light–dark cycles of host microbiomes (e.g. the human or other mammalian gut microbiome) (Jones et al., 2021; Risley et al., 2021) and aquatic microbial communities (Grubisic et al., 2017; Hörnlein & Bolhuis, 2021; Kelly et al., 2019; Silveira et al., 2017; van der Meer et al., 2007), suggesting that it is feasible and logical to assess bacterial communities in situ, such as in soils. Therefore, substantial room exists to improve our understanding of light–dark cycles in natural soil bacterial communities with potential implications for fundamental soil microbial ecology.

Accordingly, this study aimed to better understand how soil bacterial composition and network complexity changed across different land cover types and light–dark cycles. First, we hypothesised that land cover type would affect bacterial community composition, diversity (α‐ and β‐diversity) and network complexity. Second, we hypothesised that bacterial community composition (β‐diversity and taxa abundances) and networks would be different depending on the time of day because of the influence of plant–soil feedback loops and the strong effect light–dark cycles have on plants (i.e. light and temperature).

## 2 METHODS

### 2.1 Study area

**FIGURE 1:**
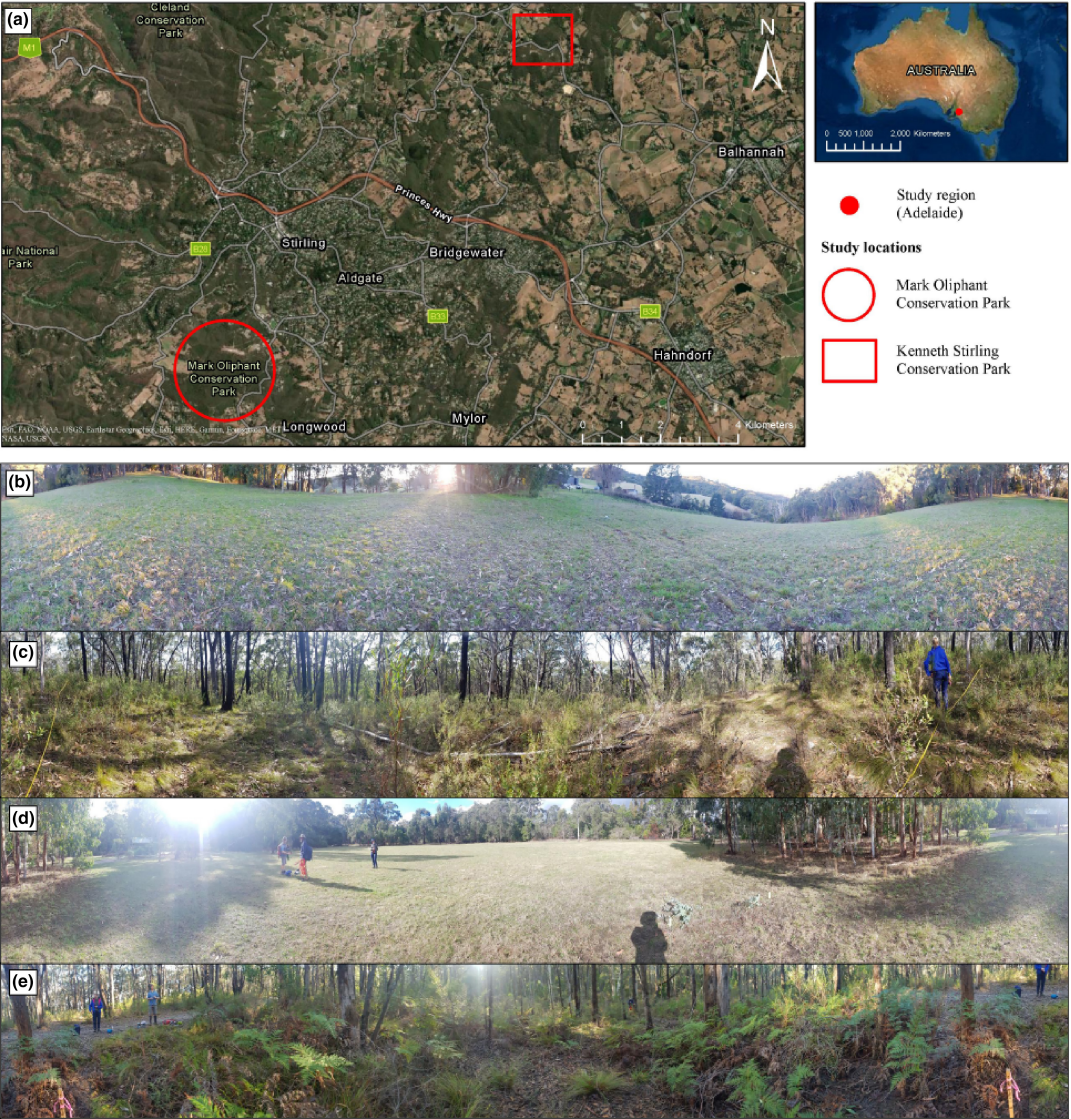
Map of the study region and panoramic photographs of the four study plots. (a) Map of the study region; (b) Cleared land cover plot at Kenneth Stirling Conservation Park; (c) Native land cover plot at the Kenneth Stirling Conservation Park; (d) Cleared land cover plot at the Mark Oliphant Conservation Park; (e) Native land cover plot at the Mark Oliphant Conservation Park. (Photo credits: Nicole Fickling).

We sampled four plots across two study sites, Mark Oliphant Conservation Park (hereafter called Mark Oliphant CP) and Kenneth Stirling Conservation Park (hereafter called Kenneth Stirling CP) in the Mount Lofty Ranges, Adelaide, South Australia, Australia, on Kaurna and Peramangk Country (Table 1; Figure 1). These sites were selected as they had the required land cover types in close proximity and both had similar pre‐European vegetation communities. Each site had adjoining and large (i.e. >1 ha) grassy and native vegetation land cover types. The grassy areas were cleared of all non‐grass vegetation (hereafter called ‘cleared’ plots) and the native woodland areas had remnant vegetation (hereafter called ‘native’ plots). We established spatially‐paired 25 m × 25 m (NSEW‐oriented) plots in each land cover type at both sites. These paired plots were less than 500 m apart within each site, with native plots situated at least 100 m away from the adjacent cleared land to reduce the edge effects (Zhao, Song, et al., 2021). We collected soil moisture and below (−10 cm) and aboveground temperatures (+0 cm, +10 cm) with TMS‐4 data loggers (TOMST, Prague, Czech Republic), which were installed at each plot for the duration of the study period (6 weeks), recording at 15‐min intervals (Wild et al., 2019).

**TABLE 1:** Plot metadata, showing the location, land cover type, vegetation metrics, soil temperature (10 cm below the ground, 0 cm above the ground and 10 cm above the ground) and soil moisture (10 cm below the ground) (soil data are the mean ± standard deviation).

Plot	Latitude (°)	Longitude (°)	Elevation (m)	Plant species richness	Canopy cover (%)	Leaf litter (%)	Soil temp −10 cm (°C)	Soil temp +0 cm (°C)	Soil temp +10 cm (°C)	Soil moisture (%)
Mark Oliphant CP Cleared	−35.03710	138.70730	369	8	0.01	0.100	9.80 ± 1.54	8.73 ± 1.54	8.36 ± 3.85	47.02 ± 1.81
Mark Oliphant CP Native	−35.03530	138.70440	370	22	27.07	26.03	9.57 ± 1.16	8.56 ± 2.43	8.28 ± 2.96	37.25 ± 3.63
Kenneth Stirling CP Cleared	−34.96686	138.77683	512	6	0.0	39.06	9.01 ± 1.07	8.35 ± 2.78	8.09 ± 2.93	50.07 ± 2.13
Kenneth Stirling CP Native	−34.96762	138.77817	512	18	29.66	27.82	8.79 ± 1.06	7.97 ± 2.12	7.88 ± 2.49	41.53 ± 2.12

### 2.2 Vegetation surveys

Vegetation surveys at each plot were done between May 24 and 26, 2022. We used an established vegetation survey approach based on White et al. (2012) with north–south facing point‐intercept transect surveys done at 0, 5, 10, 15, 20 and 25 m of each plot. At each 1 m interval within transects (*n* = 156), we recorded the plant species, growth forms and ground cover (e.g. leaf litter, bare ground) present to generate plant species richness and growth form/ground cover proportions for each plot.

### 2.3 Soil sampling

Soil samples were collected to assess bacterial communities (described below) at 6‐h intervals (00:00, 06:00, 12:00 and 18:00). We sampled across a 24‐h sampling period at each plot once per week repeated across 6 consecutive weeks from 14 June to 21 July 2022, resulting in six replicates of each site‐land cover‐time combination (*n* = 6 replicates × 2 study sites × 2 land cover plots × 4 time intervals = 96 total samples).

#### 2.3.1 Bacterial community soil samples

Soils were collected from the top 10 cm at nine grid points following the Biomes of Australian Soil Environments database protocols (Bissett et al., 2016) using a decontaminated trowel (by using 5% bleach and DECON‐90 following protocols in Cando‐Dumancela et al., 2021, 2022) and homogenised in a sterile sample bag. From this, subsamples of 50 mL were collected and stored on ice, then frozen at −20°C within 1 h of sampling.

#### 2.3.2 Physicochemical soil samples

From the composite soil samples taken for bacterial community analysis, 200 g was subsampled from all 12:00 samples (per plot *n* = 6) and sent for physicochemical analysis. Samples were analysed by CSBP Labs (Bibra Lake, WA 6163), quantifying levels of phosphorus, potassium, sulphur, organic carbon, nitrate nitrogen, ammonium nitrogen, electrical conductivity, pH and texture using protocols described on the service provider's website (https://www.csbplab.com.au/tests/soil).

### 2.4 DNA extraction, PCR, sequencing and bioinformatics

DNA extractions were completed in six batches and soil samples were processed within 1 week of the sample collection. DNA extractions were done using the Qiagen DNeasy PowerLyzer Soil Kit (Qiagen, Hilden, Germany) following the manufacturer's instructions and DNA quantification was completed using the QuantiFluor dsDNA System (Promega, Madison, WI, USA) and Quantus Fluorometer (Promega, Madison, WI, USA). The 16S rRNA gene region was targeted using the 27F and 519R primer set (Lane, 1991), PCR‐amplified and sequenced on the Illumina MiSeq platform at the Australian Genome Research Facility (AGRF; Adelaide, SA, Australia). Raw FASTQ files are available at https://www.ncbi.nlm.nih.gov/sra/PRJNA1072833.

FASTQ files were processed through the QIIME 2 bioinformatics pipeline (Bolyen et al., 2019) in a conda environment (Continuum Analytics). Due to poor‐quality reverse reads, only the forward reads were used. The *Cutadapt* plugin was used to remove primer sequences and quality‐check the raw sequences (Martin, 2011). The *Figaro* tool was used to identify the optimal trimming lengths (Sasada et al., 2020) for forward reads. These were then trimmed according to the *Figaro* output using *DADA2* (Callahan et al., 2016). Amplicon sequence variants (ASVs) were assigned using the *SILVA* version 138.1 rRNA database (Glöckner et al., 2017; Quast et al., 2013; Yilmaz et al., 2014) via the q2‐feature‐classifier (Bokulich et al., 2018) and classify‐sklearn naïve Bayes taxonomy classifier (Bokulich et al., 2018).

### 2.5 Statistical analyses

All statistics were done in R v4.1.2 (R Core Team, 2021). Soil temperature and moisture were analysed with Kruskal–Wallis tests comparing the cleared and native land cover types within sites. Physicochemical characteristics were analysed with two‐way ANOVA (where the factor land cover type is nested within the factor site), Tukey's honestly significant difference post‐hoc (to yield adjusted *p*‐values) and Kruskal–Wallis tests on the land cover type after separating sites.

#### 2.5.1 Bacterial diversity

The *phyloseq* package (McMurdie & Holmes, 2013) was used for downstream analyses of bacterial community data. Amplicon sequence variants (ASVs) were discarded if they were not classified as Bacteria, classified as ‘Mitochondria’, ‘Chloroplast’, or were present in <2 samples to avoid potential effects of contamination (Allen et al., 2016). Samples were then rarefied to the sample with the lowest read depth (Hong et al., 2022). The effective number of ASVs (alpha diversity) was calculated using the exponent of Shannon's diversity index values (Jost, 2006). Mann–Whitney *U*‐test was applied to compare bacterial alpha diversity between sites. The sites, Mark Oliphant CP and Kenneth Stirling CP, were then separated and the Mann–Whitney *U*‐test applied to analyse the effect of land cover type on bacterial alpha diversity. Within each site, land cover types were separated (i.e. Kenneth Stirling CP cleared) and Kruskal–Wallis tests were used to assess the differences in bacterial alpha diversity between the times of day. The False Discovery Rate with Benjamini‐Hochberg procedure was used to adjusted *p*‐values due to multiple comparisons (Chen et al., 2017). Rarefied samples were used to assess beta diversity between site, land–cover type and times of the day. Bray–Curtis distances were visualised in a principal coordinates analysis plot using the *ordinate* function in *phyloseq* and permutational multivariate analysis of variance (PERMANOVA) applied with a Type I sum of squares using the *adonis* function in the *vegan* package (Oksanen et al., 2022).

#### 2.5.2 Canonical correspondence analysis

Using the *ordistep* function in *vegan*, a canonical correspondence analysis (CCA) was run to analyse and visualise the associations between environmental factors and the soil bacterial community. Scaled (mean‐centred and divided by the standard deviation) soil physicochemical properties, soil temperature, soil moisture and vegetation richness were included in the analysis. Highly correlated (>0.75) variables were identified (vegetation species richness, canopy cover, ammonium nitrate and pH) and removed using the *findCorrelation* function in *caret* (Kuhn, 2021). Model‐selected soil variables were tested with permuted ANOVA with 999 permutations.

#### 2.5.3 Network analysis

To understand bacterial interactions and community structure (i.e. complexity) across sites, land cover types and the four sampling times (00:00, 06:00, 12:00, 18:00), we evaluated co‐occurrence association networks of bacterial ASVs. We constructed networks at the phylum taxonomic level to examine broad complexity and the genus level to visualise higher resolution complexity, recognising that the accuracy of species‐level associations would be low due to the high similarity between the 16S rRNA gene from closely related species. In the evaluated networks, vertices (also known as ‘nodes’) represent taxa (genera or phyla) and edges (also known as ‘links’) connect a pair of taxa if their frequencies are significantly associated (absolute abundance >0.75, *p* = <.01 for phylum and >0.95, *p* = <.01 for genus). The *p*‐values were Holm–Bonferroni corrected. The type of association, whether positive (representing a mutualistic interaction) or negative (representing an antagonistic interaction), was denoted with blue and red edges, respectively. We identified hub taxa as the bacterial taxonomic groups with the most positive and negative edges for each plot/time at the genus and phylum taxonomic level. We use the term ‘hub taxa’ to avoid potential misinterpretations associated with the term ‘keystone species’ and its ecological connotations. Our focus on elucidating indicators of key players in microbial networks rather than implying an explicit ecological role. To account for compositional bias associated with ASV data, we used *SparCC* (Friedman & Alm, 2012) to define associations. Only ASVs with sequence counts >10 were included for phylum and >100 for genus to improve visualisation, selection rigour and computational processing. Randomly permuted (*n* = 100) data were used to estimate the statistical significance of associations. We used the R package *Matrix* (Bates et al., 2023) to create a matrix from the given set of values and *igraph* (Csárdi et al., 2023) to visualise and evaluate the plots.

#### 2.5.4 Differential abundance analysis

Separately within each of the four plots (2 sites × 2 land cover types), globally differentially abundant ASVs across sampling times were identified using the *ANCOM‐BC* algorithm (Lin & Peddada, 2020) by assessing log‐fold‐changes in ASV abundances at 06:00, 12:00 and 18:00 sampling times, compared to midnight (00:00) as the intercept/baseline sampling time. We used the *ancombc()* function in the R *ANCOMBC* package () with settings including alpha = 0.05 significance level, *p*‐value adjustment for multiple comparison using the method of Holm (1979), taxa with fractional prevalence less than 0.1 (=prv_cut) were excluded in the analysis, structural zeros were detected (struc_zero = TRUE) with taxa classified as structural zeros using asymptotic lower bounds (neg_lb = TRUE) and a conservative variance estimator was used for the test statistic (conserve = TRUE). We used the *ANCOM‐BC* global test result, which identifies differentially abundant taxa between at least two groups across three or more different groups. To determine whether differentially abundant ASVs were shared across the plots, we constructed Venn diagrams to display the number of overlapping or non‐overlapping differentially abundant ASVs across plots and comparison periods (06:00 cf. 00:00, 12:00 cf. 00:00 and 18:00 cf. 00:00).

## 3 RESULTS

### 3.1 Vegetation

The vegetation communities at Mark Oliphant CP and Kenneth Stirling CP were native eucalypt woodlands with a shrubby understory. The cleared plots were characterised by graminoid and herb species (Figure S1), with a higher plant species richness in Mark Oliphant CP compared to Kenneth Stirling CP (Mark Oliphant CP: *n* = 8; Kenneth Stirling CP: *n* = 6; Figure 1b, d; Table 1). *Eucalyptus obliqua* and *E. baxteri* dominated the canopy at both native plots, with a range of small trees, shrubs, herbs and graminoids in the understory (Figure 1c, e; Figure S1). Plant species richness was greater in Mark Oliphant CP compared to Kenneth Stirling CP (Mark Oliphant CP: *n* = 22; Kenneth Stirling CP: *n* = 18; Table 1).

### 3.2 Soil temperature, moisture and physicochemical characteristics

Soil temperature and moisture data were collected at each plot at 15‐min intervals for the duration of the study. Within Kenneth Stirling CP, soil temperature 10 cm below ground was higher in the cleared land cover plot (mean ± SD; cleared: 9.01°C ± 1.07°C; native: 8.79°C ± 1.06°C; H = 77.81, *p* < .001; Table 1). The same pattern followed at Mark Oliphant CP with higher soil temperature 10 cm below ground in the cleared land cover plot (cleared: 9.80°C ± 1.54°C; native: 9.57°C ± 1.16°C; H = 58.70, *p* < .001; Table 1). Soil temperatures at 0 and 10 cm above ground followed the same patterns in both Kenneth Stirling CP and Mark Oliphant CP (Table 1). Similarly, the mean soil moisture was higher in the cleared land cover plot at Kenneth Stirling CP (cleared: 50.07% ± 2.13%; native: 41.53% ± 2.12%; H = 5924.3, *p* < .001; Table 1) and Mark Oliphant CP (cleared: 47.02% ± 1.81%; native: 37.25% ± 3.63%; H = 5689.1, *p* < .001; Table 1).

Soil physicochemical characteristics differed dramatically between plots but without clear patterns. Land cover type had a strong effect on ammonium nitrate, nitrate nitrogen, phosphorus, potassium, sulphur, organic carbon and pH. In particular, organic carbon levels were higher in the native land cover plot at Mark Oliphant CP (mean ± SD; cleared: 2.85% ± 0.14%; native: 4.12% ± 0.47%; *p*‐adj < .001, 95% CI [−1.75, −0.784]; Table 2), but were lower in the native land cover plot at Kenneth Stirling CP (cleared: 3.20% ± 0.21%; native: 2.57% ± 0.27%; *p*‐adj = .008, 95% CI: [0.139, 1.104]; Table 2). Sulphur levels were also greater in the native land cover plot at Mark Oliphant CP (cleared: 5.03 mg/kg ± 0.50 mg/kg; native: 9.00 mg/kg ± 3.00 mg/kg; *p*‐adj = .004, 95% CI [−6.71, −1.22]; Table 2) but were not different between land cover types at Kenneth Stirling CP (cleared: 6.67 mg/kg ± 1.30 mg/kg; native: 4.60 mg/kg ± 0.76 mg/kg; *p*‐adj = .185, 95% CI [−0.679, 4.81]; Table 2). Notably, potassium, phosphorus and pH levels were substantially higher in the cleared land‐cover plot at Kenneth Stirling CP compared to all other plots (Table 2).

**TABLE 2:** Soil physicochemical properties summary of land cover types within sites.

Sample	Ammonium nitrogen (mg/kg)	Nitrate nitrogen (mg/kg)	Phosphorus Colwell (mg/kg)	Potassium Colwell (mg/kg)	Sulphur (mg/kg)	Organic carbon (%)	Conductivity (dS/m)	pH level (CaCl_2_)
Kenneth Stirling Cleared	8.83 ± 1.46	3.78 ± 5.08	52.00 ± 4.62	278.17 ± 35.52	6.67 ± 1.19	3.20 ± 0.19	0.04 ± 0.01	4.88 ± 0.04
Kenneth Stirling Native	5.33 ± 2.87	10.82 ± 9.21	5.83 ± 1.07	122.83 ± 25.24	4.60 ± 0.69	2.57 ± 0.25	0.04 ± 0.01	4.58 ± 0.07
Mark Oliphant Cleared	9.67 ± 1.60	19.50 ± 6.34	5.67 ± 0.47	121.17 ± 7.38	5.03 ± 0.46	2.85 ± 0.12	0.05 ± 0.01	4.65 ± 0.08
Mark Oliphant Native	20.50 ± 14.06	1.78 ± 1.25	4.67 ± 0.94	108.33 ± 15.25	9.00 ± 2.74	4.12 ± 0.43	0.06 ± 0.01	4.50 ± 0.12

*Note*: Values are mean ± standard deviation of the raw/untransformed data.

### 3.3 Bacterial community and diversity

**FIGURE 2:**
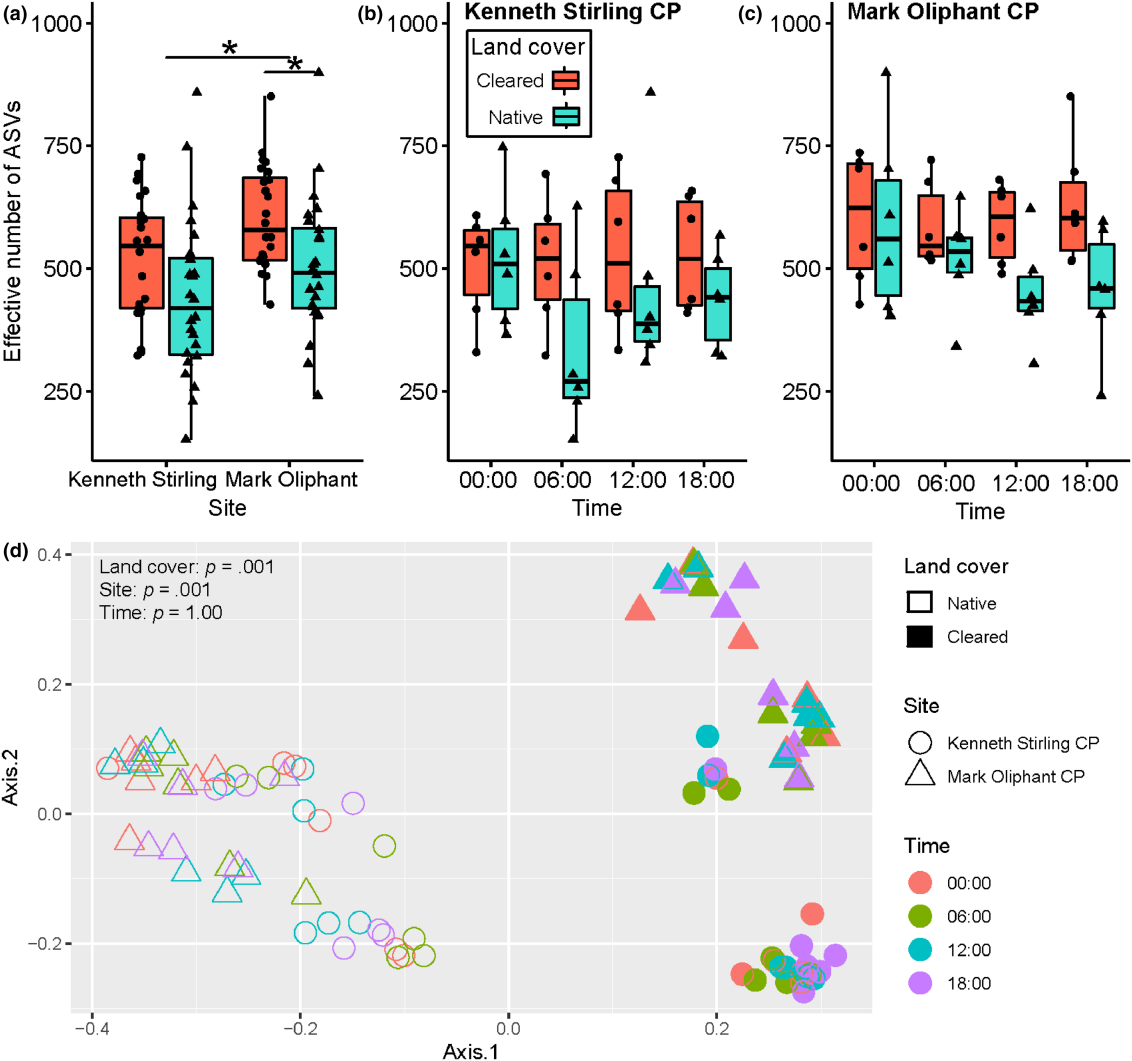
16S rRNA amplicon sequencing results. Cleared land cover plots are shown in red and native land cover plots are shown in turquoise. Effective number of ASVs between (a) sites/land cover, (b) times at Kenneth Stirling CP and (c) times at Mark Oliphant CP. Principal coordinates analysis plot using Bray–Curtis distance (d). *p < .01.

After cleaning and filtering of sequence data, a total of 12,173 bacterial amplicon sequence variants (ASVs) were detected across the 96 samples (Table S1). Sample reads were rarefied to the lowest read count of 23,073 reads (from #30 sample—KSN 00:00 week 6; Table S1). The effective number of ASVs was calculated to compare alpha diversity between sites, land cover and times. Alpha diversity at Mark Oliphant CP was higher than Kenneth Stirling CP (mean ± SD, Mark Oliphant CP: 554 ± 132 effective number of ASVs; Kenneth Stirling CP: 479 ± 149 effective number of ASVs; *U* = 6.28, *p*‐adj = .042; Figure 2a). There was also strong evidence that alpha diversity was higher at the cleared land cover type at Mark Oliphant CP (cleared: 603 ± 104 effective number of ASVs; native: 504 ± 139 effective number of ASVs; *U* = 8.82, *p*‐adj = .018; Figure 2a) but only weak evidence at Kenneth Stirling CP (cleared: 519 ± 123 effective number of ASVs; native: 439 ± 164 effective number of ASVs; *U* = 4.42, *p*‐adj = .083).

Time did not have an effect on alpha diversity in Kenneth Stirling CP at either the cleared (mean ± SD; 00:00: 505 ± 109 effective number of ASVs; 06:00: 513 ± 132 effective number of ASVs; 12:00: 529 ± 161 effective number of ASVs; 18:00: 529 ± 119 effective number of ASVs; *H* = 0.313, *p*‐adj = .957; Figure 2b) or native land cover plot (00:00: 520 ± 140 effective number of ASVs; 06:00: 339 ± 180 effective number of ASVs; 12:00: 462 ± 203 effective number of ASVs; 18:00: 436 ± 99.4 effective number of ASVs; *H* = 4.547, *p*‐adj = .364; Figure 2b). Time also had no effect on alpha diversity at Mark Oliphant CP at the cleared (00:00: 602 ± 134 effective number of ASVs; 06:00: 589 ± 88.3 effective number of ASVs; 12:00: 591 ± 82 effective number of ASVs; 18:00: 631 ± 127 effective number of ASVs; *H* = 0.393, *p*‐adj = .957; Figure 2c) or native land cover type (00:00: 591 ± 188 effective number of ASVs; 06:00: 518 ± 103 effective number of ASVs; 12:00: 450 ± 105 effective number of ASVs; 18:00: 457 ± 129 effective number of ASVs; *H* = 2.61, *p*‐adj = .639; Figure 2c).

**FIGURE 3:**
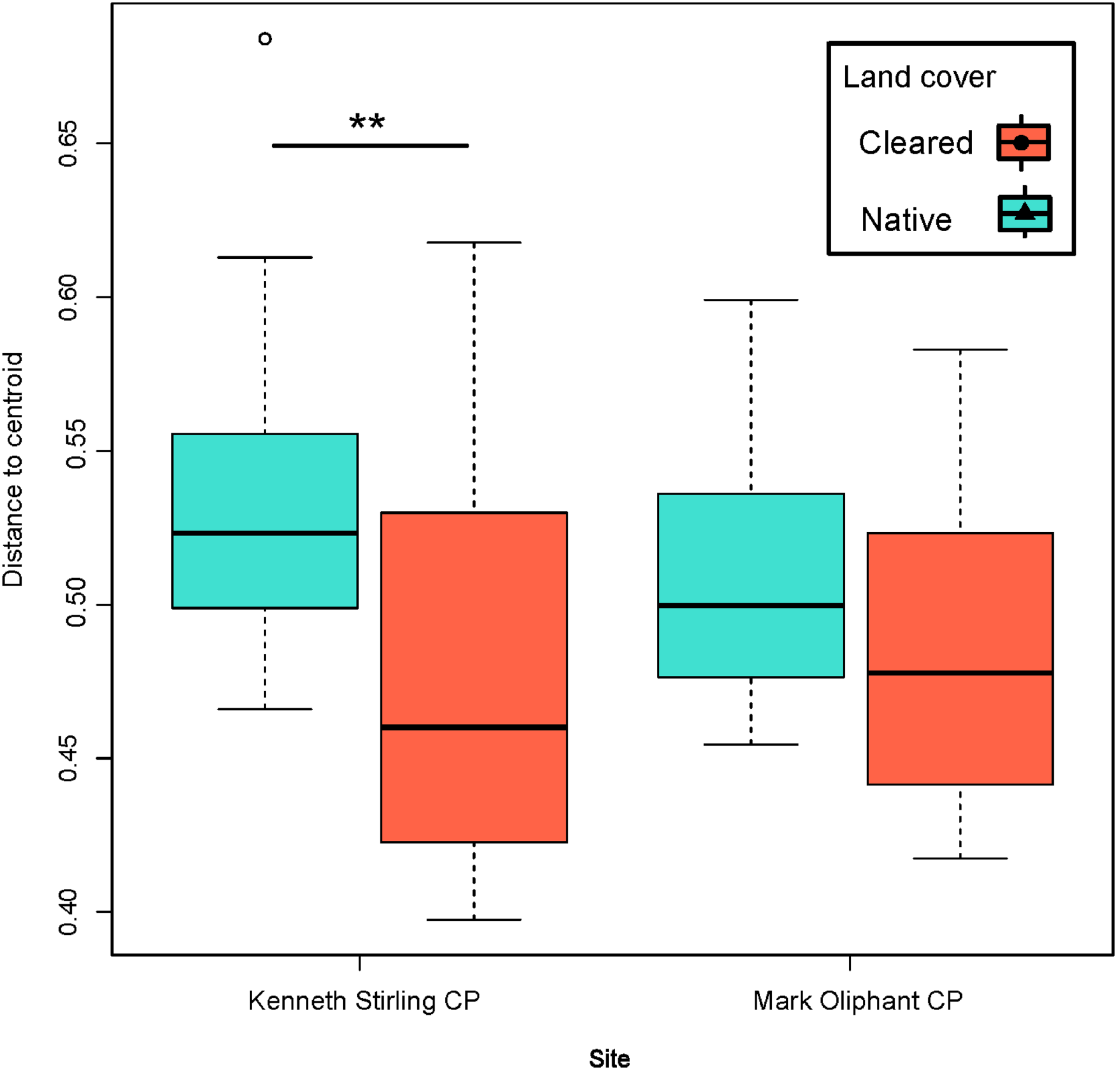
Distance to centroid of samples comparing site/land cover types. Cleared land cover plots are indicated in red and native land cover plots indicated in turquoise. Distance to centroid calculated on the Bray–Curtis distance in a principal coordinates analysis space. **p < .001.

The principal coordinate analysis and PERMANOVA based on Bray–Curtis distance showed that land cover (*p* = .001) had a stronger and significant effect on beta diversity than site (*p* = .001) and time (*p* = 1.0) (Figure 2d). The site effect was stronger in the cleared land cover types, with Kenneth Stirling CP and Mark Oliphant CP samples separating more distinctly than in the native land cover (Figure 2d). There was also a greater bacterial community heterogeneity measured by distance to the centroid in the native land cover plot compared to the cleared land cover plot in Kenneth Stirling CP (mean ± SD; cleared: 0.477 ± 0.062 distance to centroid; native: 0.534 ± 0.0456 distance to centroid; *p*‐adj = .001, 95% CI [−0.096, −0.018]; Figure 3). The same trend was seen in land cover at Mark Oliphant CP though this was not significant (cleared: 0.483 ± 0.046; native: 0.508 ± 0.040; *p*‐adj = .351, 95% CI [−0.063, −0.014]; Figure 3).

### 3.4 Canonical correspondence analysis

**FIGURE 4:**
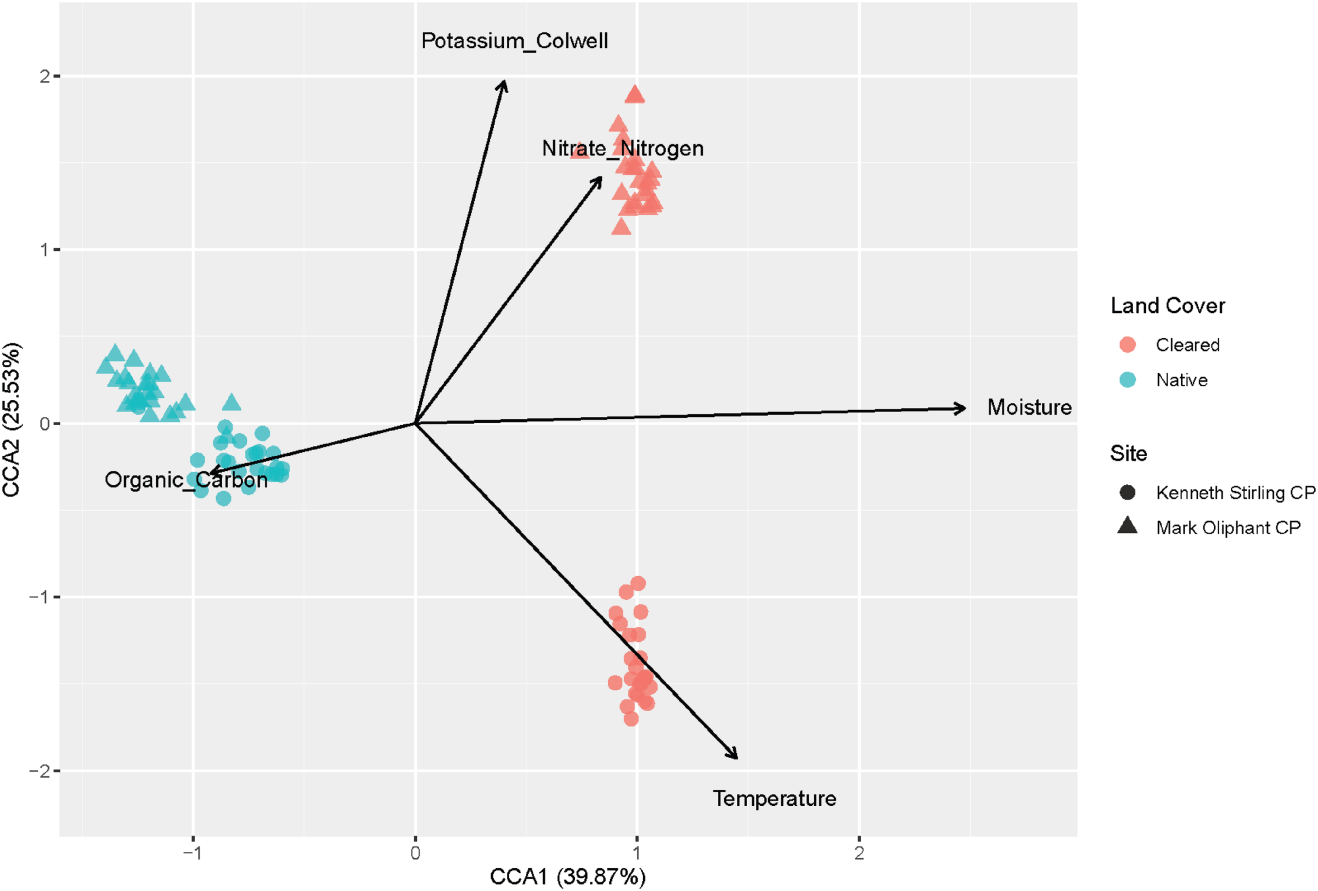
Canonical correspondence analysis on the associations between environmental and edaphic factors with the microbial community. Cleared land cover plots are indicated in red and native land cover plots in blue. Kenneth Stirling CP samples are represented by cirlces and Mark Oliphant CP samples are represented by triangles.

A canonical correspondence analysis (CCA) was run to analyse the associations between environmental factors and the soil bacterial community. The CCA model selection identified soil moisture, temperature, nitrate nitrogen, potassium and organic carbon as being associated with the bacterial community composition and ANOVA showed strong support for this model (df = 5, *F* = 2.940, *p* = .001) (Figure 4). Soil moisture had a strong association with the soil bacterial community driving the communities across land cover types.

### 3.5 Bacterial networks

**FIGURE 5:**
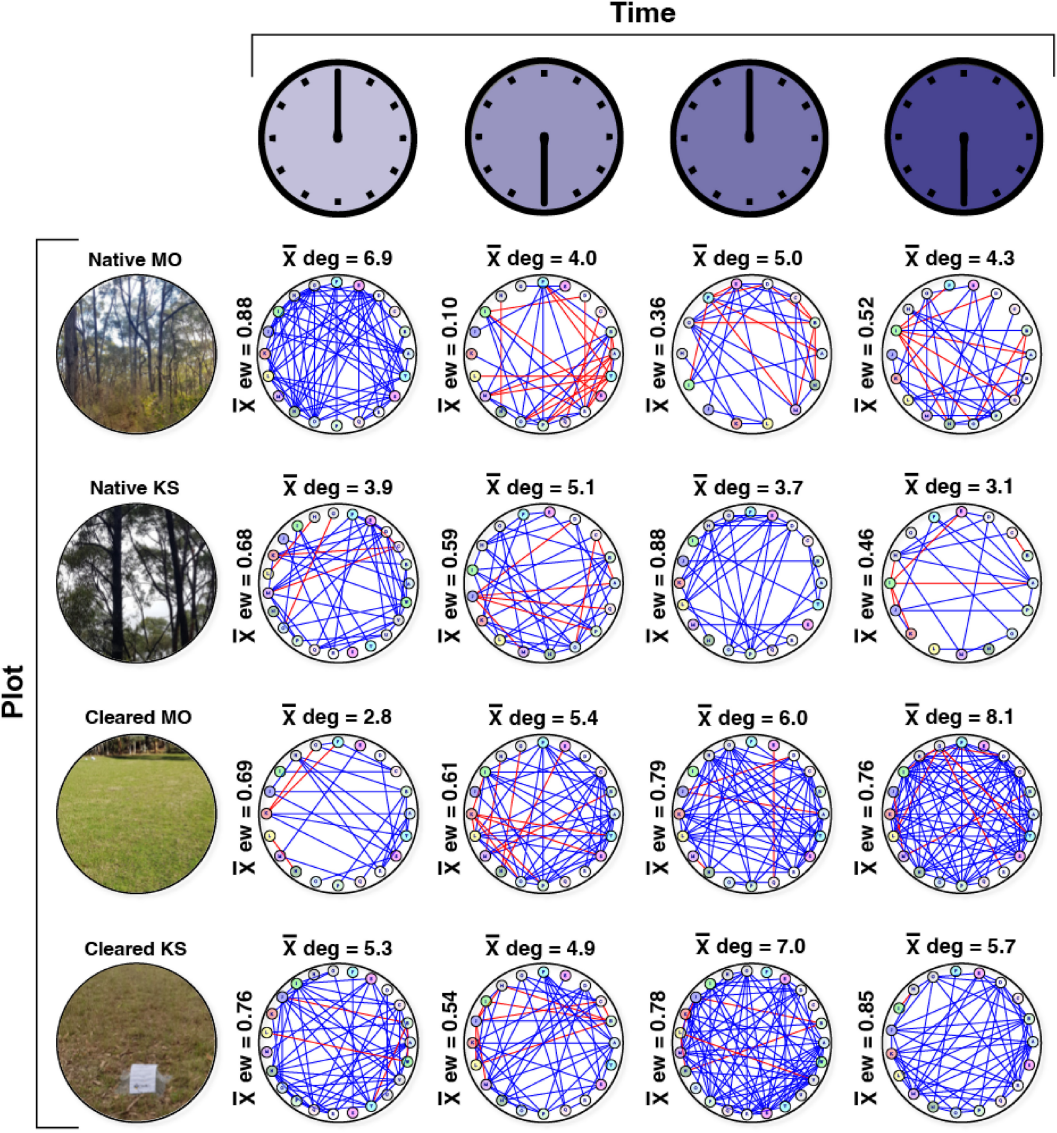
Association network plots of bacterial phyla for the four plots (Native MO, Native KS, Cleared MO and Cleared KS) and sampling times (00:00, 06:00, 12:00 and 18:00). Vertex colour represents phyla (see Supporting Information for more information). Blue edges represent positive associations and red edges represent negative associations. The mean degree is shown above each network and the mean edge weight (ew) is to the left of each network. MO, Mark Oliphant CP; KS, Kenneth Stirling CP.

**FIGURE 6:**
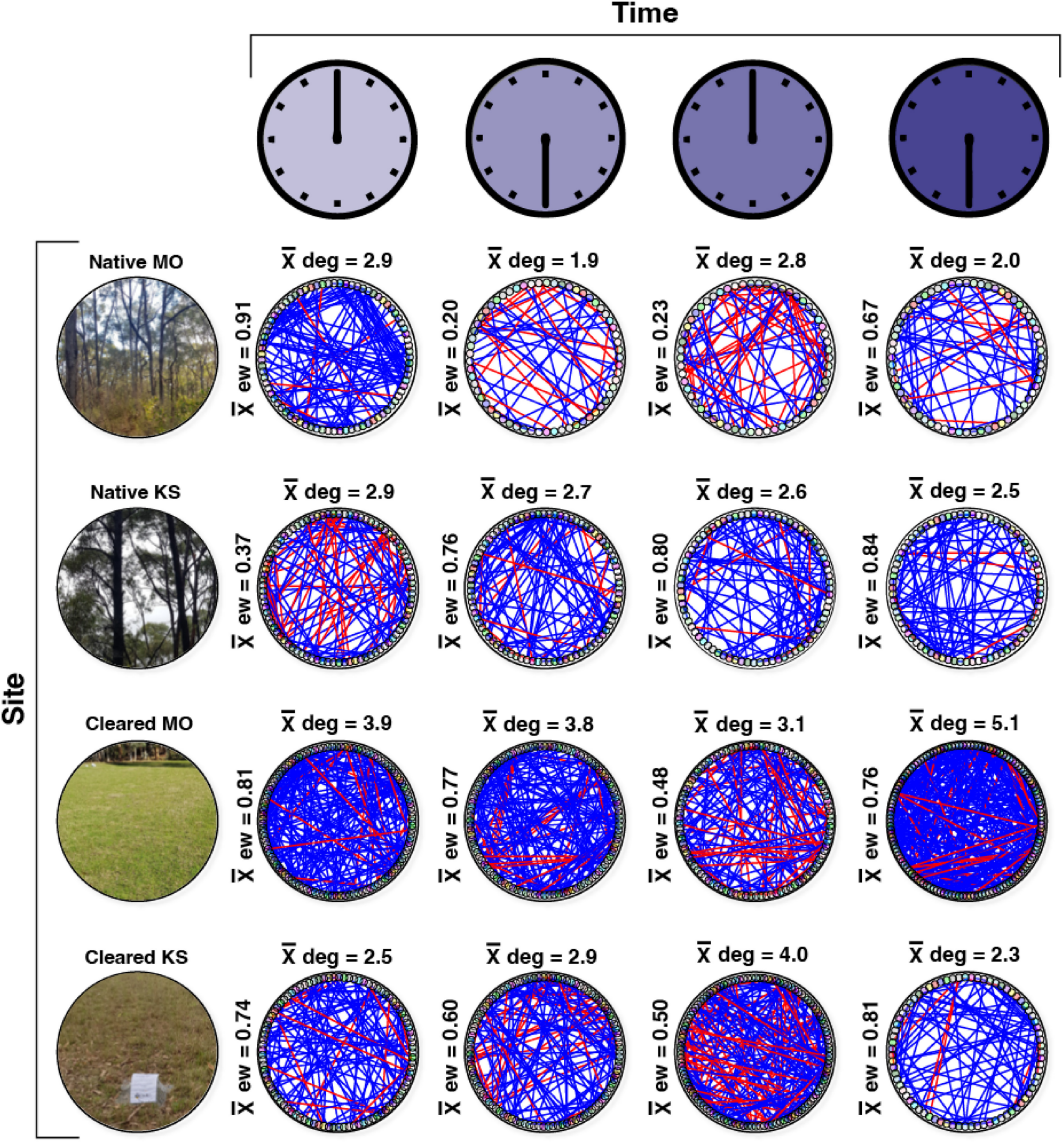
Association network plots of bacterial genera for the four plots (Native MO, Native KS, Cleared MO and Cleared KS) and sampling times (00:00, 06:00, 12:00 and 18:00). Vertex colour represents genera (see Supporting Information for more information). Blue edges represent positive associations and red edges represent negative associations. The mean degree is shown above each network and the mean edge weight (ew) is to the left of each network. MO, Mark Oliphant CP; KS, Kenneth Stirling CP.

The association network analyses highlighted differences in community complexity and interactions across sampling times and land cover types, defined by the node degree (the average number of edges connecting the vertices), network size and edge weight (i.e. whether the interactions were positive or negative) (Figures 5 and 6; Table S2).

Bacterial phyla in the cleared plots had a higher node degree (total $\bar{x}$ node degree = 45.2) than the native plots (total $\bar{x}$ node degree = 36) (*t* = −3.18, df = 148, *p* = <.01) (Figure 5). There were also clear differences in the node degree across sampling times. The Mark Oliphant CP cleared plot had a steadily increasing node degree from 00:00 through to 18:00. Moreover, the mean node degree was typically higher for native plots between 00:00 and 06:00 (combined $\bar{x}$ node degree = 19.9) than between 12:00 and 18:00 (combined $\bar{x}$ node degree = 16.1) (*t* = 2.22, df = 80, *p* = .02) but was generally lower for cleared plots between 00:00 and 06:00 (combined $\bar{x}$ node degree = 18.4) than between 12:00 and 18:00 (combined $\bar{x}$ node degree = 26.8) (*t* = −4.36, df = 83, *p* = <.01). Evaluation of either positive or negative edge types highlighted differences across land cover types with more negative associations among phyla in the native plots. One striking finding was that the mean edge weight between 00:00 and 06:00 in the native Mark Oliphant CP plot dropped from 0.88 (a high level of positive interactions) to 0.10 (a high level of negative interactions).

Bacterial genera in the cleared plots had a higher node degree (total $\bar{x}$ node degree = 27.6) than the native plots (total $\bar{x}$ node degree = 20.3) (*t* = 021.5, df = 3099, *p* = <.001) (Figure 6), as expected following the phylum‐level analysis. There were also clear differences in the node degree across sampling times; however, the patterns were variable. The native Kenneth Stirling CP plot had a steadily decreasing node degree from 00:00 through to 18:00. The cleared Kenneth Stirling CP plot had a steadily increasing node degree from 18:00 to 12:00. There were also differences in edge weight trends across site types with more negative associations among genera in the native plots (combined $\bar{x}$ edge weight = 4.78) than cleared plots (combined $\bar{x}$ edge weight = 5.47), approaching conventional significance (*t* = 1.8, df = 7, *p* = .056). The striking difference in mean edge weight between 00:00 and 06:00 in the native Mark Oliphant CP plot was also visible in the genus‐level network plots. Conversely, the mean edge weight *increased* between native Kenneth Stirling CP plots at 00:00 and 06:00.

The evaluation of hub taxa (bacterial groups with the highest degree of either positive or negative associations) showed that the bacteria at the genus level were different at each sampling time for both the native and cleared plots, with positive degree ranging from 4 to 19 and negative degree ranging from 0 to 15 (Table 3). At the phylum level, Acidobacteriota were the most common phylum with the highest number of positive associations, occurring in native Kenneth Stirling and Mark Oliphant CP plots at 18:00 and the cleared Mark Oliphant CP plot at 06:00 and 12:00. Bdellovibrionota were the top negative hub phylum, featuring in native Kenneth Stirling CP 00:00, native Mark Oliphant CP 12:00 and cleared Mark Oliphant CP 12:00.

**TABLE 3:** Bacterial taxonomic groups with the most positive and negative edges for each plot/time.

Plot/time	Top positive taxa	Top negative taxa	Positive degree	Negative degree
Phylum				
Native KS 00:00	Bacteroidota	Bdellovibrionota	9	5
Native KS 06:00	Cyanobacteria	SAR324_clade	6	9
Native KS 12:00	Planctomycetota	N/A	8	N/A
Native KS 18:00	Acidobacteriota	Firmicutes (Bacillota)	7	6
Native MO 00:00	Dependentiae	N/A	12	N/A
Native MO 06:00	SAR324_clade	Proteobacteria	9	8
Native MO 12:00	Armatimonadota	Bdellovibrionota	6	8
Native MO 18:00	Verrucomicrobiota	Firmicutes (Bacillota)	7	7
Cleared KS 00:00	Patescibacteria	Elusimicrobiota	11	10
Cleared KS 06:00	Acidobacteriota	Myxococcota	9	6
Cleared KS 12:00	Gemmatimonadota	Latescibacterota	12	5
Cleared KS 18:00	Bdellovibrionota	N/A	10	N/A
Cleared MO 00:00	Fibrobacterota	Planctomycetota	5	2
Cleared MO 06:00	Acidobacteriota	Latescibacterota	10	4
Cleared MO 12:00	Acidobacteriota	Bdellovibrionota	11	1
Cleared MO 18:00	Elusimicrobiota	Methylomirabilota	13	3
Genus				
Native KS 00:00	*Armatimonadales*	*Nakamurella*	6	11
Native KS 06:00	*Caulobacter*	*CPla‐3 termite group*	6	10
Native KS 12:00	*Tundrisphaera*	*Gaiella*	7	7
Native KS 18:00	*Pseudomonas*	*Actinomycetospora*	7	6
Native MO 00:00	*Vicinamibacteraceae*	*TK10*	11	1
Native MO 06:00	*Jatrophihabitans*	*Inquilinus*	4	7
Native MO 12:00	*Aquisphaera*	*Granulicella*	13	5
Native MO 18:00	*TRA3‐20*	*Subgroup_7*	5	4
Cleared KS 00:00	*RB41 (Acidobacter)*	*Jatrophihabitans*	7	5
Cleared KS 06:00	*CPla‐3 termite group*	*Occapllatibacter*	11	6
Cleared KS 12:00	*WPS‐2*	*Subgroup_7*	12	14
Cleared KS 18:00	*Streptomyces*	*Kribbella*	6	3
Cleared MO 00:00	*Geodermatophilus*	*Nakamurella*	13	10
Cleared MO 06:00	*Anaeromyxobacter*	*Granulicella*	10	15
Cleared MO 12:00	*A0839*	*Candidatus*	10	11
Cleared MO 18:00	*vadinHA49*	*Obscuribacteraceae*	19	13

*Note*: The top positive and negative degrees are shown for each taxon and the table includes the results for the phylum and genus levels.

### 3.6 Differentially abundant ASVs

**FIGURE 7:**
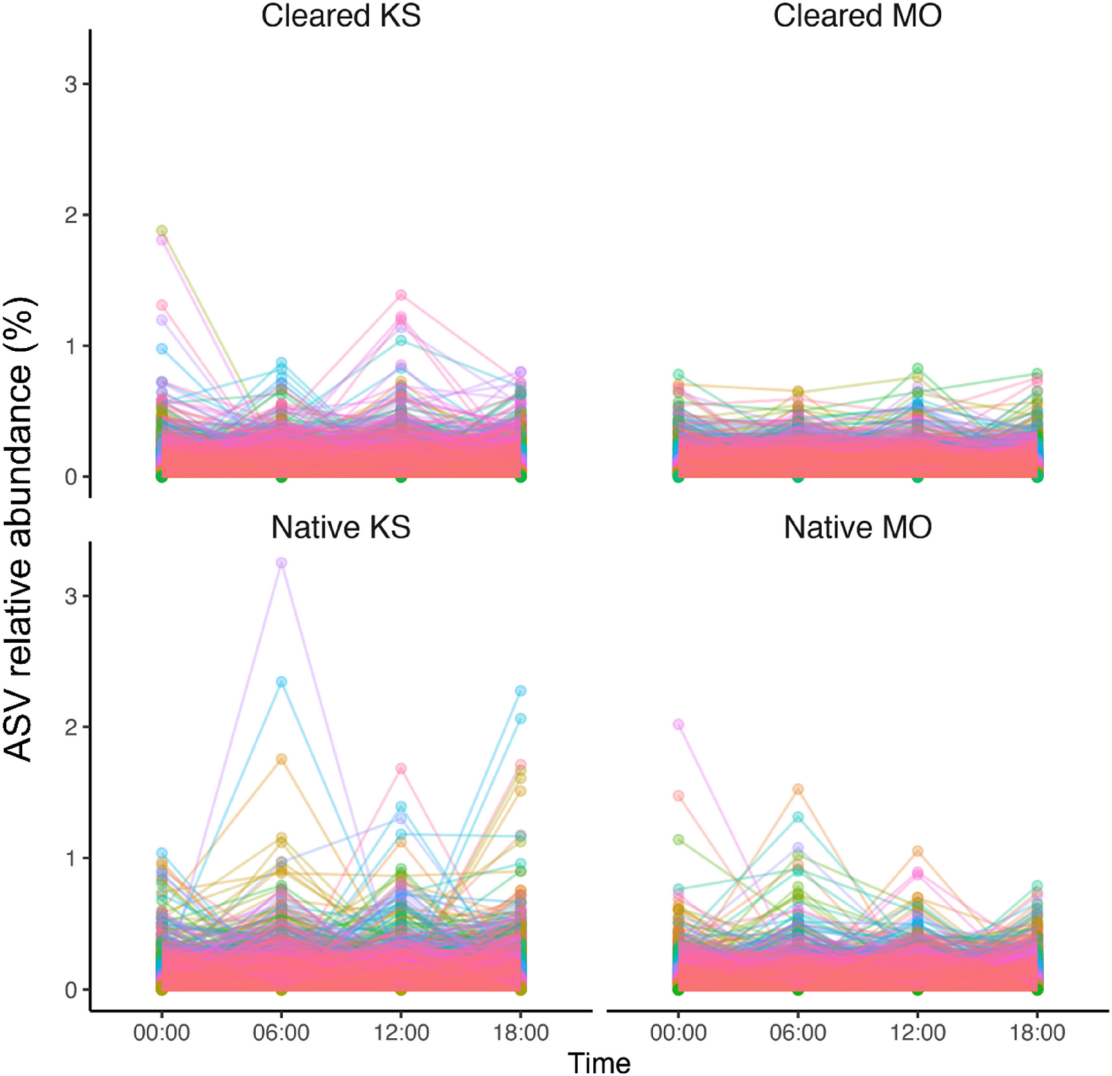
Time‐series changes in relative abundance (%) of ASVs in the four plots (Cleared KS, Cleared MO, Native KS, Native MO). Lines connect ASVs that have changed in relative abundance over time. The top 50 ASVs that fluctuate most from the midnight (00:00) samples are presented in the Supporting Information (Figures S2–S5). MO, Mark Oliphant CP; KS, Kenneth Stirling CP.

Across all the plots, sample ASV relative abundances (%) and corresponding log‐fold‐change results from ANCOM‐BC differential abundance testing were highlighted for the top 50 ASVs in each plot that displayed the largest absolute magnitudes of log‐fold change between comparison groups (see Figures S2–S5; Tables S3–S6). These most fluctuating ASVs were predominantly from the phyla Acidobacteriota, Actinobacteriota and Proteobacteria (Tables S3–S6). Respective total numbers of ASVs from each plot with at least one occasion of significant differential abundance at later measurement times (06:00, 12:00, 18:00) compared to the midnight (00:00) baseline/intercept were 853 ASVs at the cleared Kenneth Stirling CP, 598 ASVs at native Kenneth Stirling CP, 922 ASVs at cleared Mark Oliphant CP and 685 ASVs at the native Mark Oliphant CP plot. Time‐series changes in the relative abundance (%) of these differentially abundant ASVs over the sampling times were also visualised for each site (Figure 7). Non‐trivial cohorts of ASVs appeared to be varying temporally over the 24 h, and these fluctuating ASVs were largely plot‐specific (see Venn diagrams, Figure S6). Within a plot, numbers typically in the range of 200–400 ASVs were either increasing or decreasing when comparing baseline midnight (00:00) samples to later measurement times (06:00, 12:00, 18:00). Very few of the ASVs displaying temporal differential abundance were shared across plots, with approximately 1%–4% of all differentially abundant (or in the order of 10–50 ASVs) shared between sites.

## 4 DISCUSSION

We examined changes in soil bacterial communities with land cover type and light–dark cycles. We investigated these relationships by repeatedly sampling soil at two spatially‐paired plots at two South Australian conservation parks (four plots in total) over 6 weeks. From these soils, we characterised the bacterial community via 16S rRNA amplicon sequencing and generated edaphic physicochemical data (e.g. organic carbon, pH, sulphur, phosphorus). We showed that land cover type and associated soil variables strongly affected soil bacterial diversity and composition. Soils under native vegetation hosted different bacterial communities and expressed greater heterogeneity compared to soils under cleared vegetation. We also report time‐dependent and site‐specific changes in bacterial network complexity and relative abundance of many ASVs. Our results demonstrate for the first time that light–dark cycles do subtly affect the composition of soil bacterial communities in situ.

### 4.1 Effect of land cover type on the soil bacterial diversity

We show that soil bacterial alpha diversity was generally higher in the cleared land cover plots but that these plots had less heterogeneous bacterial communities—results that are consistent with previous work on the effects of urban versus natural land cover on soil bacteria. The global study by Delgado‐Baquerizo et al. (2021) showed that soil bacterial communities from human‐impacted locations had consistently higher alpha diversity compared to their paired natural ecosystem locations. Similar results were found by Han et al. (2021), comparing different vegetation communities in more urban and natural areas. Delgado‐Baquerizo et al. (2021) also showed that bacterial communities were more homogenous across urban and/or more human‐impacted soils. They suggested similarities in urban soil management practices and land cover changes primarily drove this homogeneity. In our case, both cleared land cover plots have been historically impacted by humans through the clearing of all non‐grass vegetation and continued mowing. However, our results suggested that fertilisation likely occurred only at one of the cleared land cover types, with nitrate nitrogen driving differences between bacterial communities of the two sites. Additionally, we found that soil temperatures and moisture were greater in the cleared plots than in the native plots, a pattern similarly noted by Delgado‐Baquerizo et al. (2021). Soil bacteria are generally sensitive to changes in soil temperature and moisture (Wu et al., 2015), which may influence the soil bacterial communities (Blazewicz et al., 2020; Delgado‐Baquerizo et al., 2021). This trend was supported by our results, with soil moisture being a major driver of the soil bacterial communities between land cover types. The presence of distinct soil bacterial communities in different land cover types highlights the need to not only conserve soil bacterial biodiversity present in our natural areas but also to support the development of ways to restore soil bacteria in modified areas for soil biodiversity conservation.

Similarly, our network analysis provided supporting evidence that soil bacterial community complexity was affected by land cover type. We saw fewer bacterial interactions and network complexity (lower connectance) in our native plots. Moreover, we found more positive interactions in our cleared plots and more negative interactions in the native plots. More positive associations in the cleared plots suggest these bacteria may undergo greater cooperation for resources or a lack of competition among the interacting bacteria. The results of our ‘hub taxa’ analysis showed that the bacteria with the highest node degree at the genus level were different at each land cover type. This suggests that the hub taxa have only a fleeting influence on the network structure across time. However, when examining the hub taxa at the phylum level, we found that Gram‐negative Acidobacteriota groups were most prevalent, featuring in the top position (highest node degree for positive interactions) twice for both land cover types. This suggests that Acidobacteriota may have a mutualistic role in the rhizosphere. However, it should be noted that network analyses are correlative and additional functional studies are required to confirm this. Nonetheless, our results are consistent with the literature, which confirms Acidobacteriota roles in C‐ and N‐cycling and plant health, among other functions (Huber et al., 2022; Kalam et al., 2020). These ecological functions could conceivably influence rhizosphere bacterial interactions. Regarding negative hub taxa (bacterial groups with an antagonistic association), Bdellovibrionota had the highest node degree for negative interactions, featuring twice in each land cover class. These bacteria are often obligate aerobic predators (Ortiz et al., 2021), consuming Gram‐negative bacteria, which could potentially help explain their negative association in our networks.

### 4.2 Effect of light–dark cycles on soil bacterial communities

Here, we detected numerous differentially abundant ASVs that displayed fluctuating patterns in ASV relative abundance and differences in network complexity over the 24‐h sampling period. We found that mean node degree (connectance) was significantly higher between 00:00 and 06:00 than between 12:00 and 18:00 for the native plots but significantly lower for the cleared sites between these times. This indicates a level of time‐dependent changes in bacterial network complexity with inter‐site variability. In other words, it appears that native site bacterial interactions increased in the early morning hours when it is darker (and colder) and decreased in the afternoon/evening when it is lighter, and vice versa for the cleared plots. Further, the ‘hub taxa’ analysis showed that the bacteria with the highest node degree at the genus level differed at different sampling times. These findings support the notion that light–dark cycles mediate changes in bacterial interactions (e.g. driven by the time‐dependent rhizosphere activity). One possible explanation for this light–dark cycling could be the transmission of biological rhythms from plants to the soil bacteria (Newman et al., 2022), as plants alter the physicochemical properties of the soil. Soil temperature, moisture and respiration also vary diurnally (Hu et al., 2016), which could affect these microbial interactions, as could methane fluxes via plant exudates or other microorganisms (Subke et al., 2018). The time‐dependent changes observed in bacterial network complexity and ASV relative abundances suggest that the time of sampling should be considered in soil microbial studies. The differences in bacterial interactions across land cover types between day and night are equally as interesting. This inter‐site variation suggests that vegetation complexity may influence rhizosphere microbial community interactions in combination with light–dark cycles. More research is needed to determine the drivers of this variation. However, we can speculate that vegetation community‐mediated differences in soil biogeochemistry between the more complex remnant vegetation (i.e. our native plots) and the less diverse lawn (i.e. our cleared plots) sites—resulting from factors such as variation in transpiration, shade, exudation and pH—may be responsible.

We did not observe a strong effect of time or light–dark cycles on the soil bacterial alpha or beta diversity at the community level in any plot. We are aware of no studies that have focused on characterising the light–dark cycles of soil bacterial communities in situ and to our knowledge, very few have used DNA‐based approaches to study soil bacterial light–dark cycles. Landesman et al. (2019) was the only study prior to ours that had considered the effect of light–dark cycles on bacterial communities; however, their primary focus was on seasonal variation. Light–dark patterns in bacterial communities were observed in the rhizospheres of *Arabidopsis thaliana* and rice in a range of ex situ greenhouse studies (Lu et al., 2021; Staley et al., 2017; Zhao et al., 2022; Zhao, Ma, et al., 2021). It is possible that our study did not detect a light–dark effect at the sample community level because the soils were pooled at the plot level and did not specifically target root rhizospheres (separate from bulk soils), as per the studies mentioned above. While there is variation in the life cycles of different bacterial taxa, the bacterial turnover rates in bulk soils (further away from the rhizosphere) are expected to be slower and therefore may not vary over such short temporal scales (Joergensen & Wichern, 2018; Sokol et al., 2022). Additionally, 16S rRNA amplicon sequencing may not effectively detect change over a short interval due to the stability of DNA and the inability to differentiate between live and dead bacteria (Li et al., 2017). However, as used in some greenhouse experiments (Baraniya et al., 2018; Dai et al., 2022; Staley et al., 2017), transcriptomic approaches may reveal new insights into the light–dark cycles of bacterial community activity and plant exudates in vegetation community‐wide studies. Future studies could apply these transcriptomic approaches to understand soil bacterial community activity levels rather than focusing on community composition.

## 5 CONCLUSIONS

Soil bacterial communities can be influenced by many factors (Sokol et al., 2022). We highlight the well‐studied impact of land‐cover type on soil bacterial composition, diversity and network connectedness. These findings highlight the need for further improved ecosystem restoration practices that target the soil microbiome, which is a nascent field of research (Farrell et al., 2020; Mohr et al., 2022). Our study also provides a new look into soil microbial ecology by observing in situ changes in soil bacterial communities across light–dark cycles, which is a new lens through which to study soil microbial ecology. Future soil bacteria studies using DNA‐based tools should improve the control of sampling time to avoid introducing unwanted noise into their soil bacterial datasets.

## AUTHOR CONTRIBUTIONS

**Nicole W. Fickling:** Conceptualization (equal); formal analysis (equal); investigation (lead); methodology (equal); project administration (equal); writing – original draft (lead); writing – review and editing (equal). **Catherine A. Abbott:** Conceptualization (equal); methodology (equal); supervision (supporting); writing – review and editing (equal). **Joel E. Brame:** Conceptualization (equal); investigation (supporting); methodology (equal); supervision (supporting); writing – review and editing (equal). **Christian Cando‐Dumancela:** Investigation (supporting); methodology (equal); resources (lead); writing – review and editing (equal). **Craig Liddicoat:** Formal analysis (equal); visualization (equal); writing – review and editing (equal). **Jake M. Robinson:** Formal analysis (equal); visualization (equal); writing – review and editing (equal). **Martin F. Breed:** Conceptualization (equal); funding acquisition (lead); methodology (equal); project administration (equal); supervision (lead); writing – review and editing (equal).

## CONFLICT OF INTEREST STATEMENT

The authors declare that there are no competing interests.

## BENEFIT‐GENERATED

Benefits from this research accrue from the sharing of our data and results on public databases as described above.

## Supporting information


Data S1.

## Data Availability

Raw FASTQ files are available on the Sequence Read Archive (SRA) (https://www.ncbi.nlm.nih.gov/sra/PRJNA1072833). Project data, metadata and code are available on figshare (https://doi.org/10.25451/flinders.c.6683690).
