# Supplementary material for: Light–dark cycles may influence in situ soil bacterial networks and diurnally‐sensitive taxa
Source: Ecol Evol. 2024 Feb 13;14(2):e11018. doi: 10.1002/ece3.11018 (PMC10864733; doi:10.1002/ece3.11018)
Supplement: Supplementary file 1 — Data S1. [file ECE3-14-e11018-s001.docx]

**Supplementary Material**

for the article:

**Light-dark cycles may influence *in situ* soil bacterial networks and diurnally-sensitive taxa**

Nicole W. Fickling, Catherine A. Abbott, Joel E. Brame, Christian Cando-Dumancela, Craig Liddicoat, Jake M. Robinson, and Martin F. Breed

Correspondence to: nicole.fickling@flinders.edu.au

This Supplementary Material file includes:

- Tables S1-S6
- Figures S1-S6

**Table S1.** Number of reads and amplicon sequence variants (ASVs) detected in each soil sample. MO = Mark Oliphant CP; KS = Kenneth Stirling CP; C = cleared land cover type; N =native land cover type.

| Sample # | Site/land cover | Time | Week | # of reads | # of ASVs |
| --- | --- | --- | --- | --- | --- |
| 1 | KSN | 00:00 | 1 | 37197 | 746 |
| 2 | KSN | 00:00 | 2 | 49820 | 876 |
| 3 | KSN | 00:00 | 3 | 50733 | 732 |
| 4 | KSN | 00:00 | 4 | 52473 | 1020 |
| 5 | KSN | 00:00 | 5 | 33479 | 545 |
| 6 | KSN | 00:00 | 6 | 26179 | 539 |
| 7 | KSN | 06:00 | 1 | 31209 | 653 |
| 8 | KSN | 06:00 | 2 | 45440 | 876 |
| 9 | KSN | 06:00 | 3 | 24510 | 347 |
| 10 | KSN | 06:00 | 4 | 33064 | 369 |
| 11 | KSN | 06:00 | 5 | 26771 | 389 |
| 12 | KSN | 06:00 | 6 | 27461 | 421 |
| 13 | KSN | 12:00 | 1 | 27564 | 549 |
| 14 | KSN | 12:00 | 2 | 35995 | 678 |
| 15 | KSN | 12:00 | 3 | 102974 | 1327 |
| 16 | KSN | 12:00 | 4 | 31377 | 480 |
| 17 | KSN | 12:00 | 5 | 34493 | 503 |
| 18 | KSN | 12:00 | 6 | 28259 | 533 |
| 19 | KSN | 18:00 | 1 | 35082 | 630 |
| 20 | KSN | 18:00 | 2 | 40715 | 631 |
| 21 | KSN | 18:00 | 3 | 50891 | 787 |
| 22 | KSN | 18:00 | 4 | 56012 | 858 |
| 23 | KSN | 18:00 | 5 | 29565 | 474 |
| 24 | KSN | 18:00 | 6 | 24770 | 467 |
| 25 | KSC | 00:00 | 1 | 34834 | 807 |
| 26 | KSC | 00:00 | 2 | 44391 | 811 |
| 27 | KSC | 00:00 | 3 | 53028 | 902 |
| 28 | KSC | 00:00 | 4 | 57415 | 714 |
| 29 | KSC | 00:00 | 5 | 40017 | 807 |
| 30 | KSC | 00:00 | 6 | 23073 | 505 |
| 31 | KSC | 06:00 | 1 | 35131 | 825 |
| 32 | KSC | 06:00 | 2 | 44162 | 967 |
| 33 | KSC | 06:00 | 3 | 51456 | 776 |
| 34 | KSC | 06:00 | 4 | 52210 | 871 |
| 35 | KSC | 06:00 | 5 | 24915 | 481 |
| 36 | KSC | 06:00 | 6 | 30784 | 629 |
| 37 | KSC | 12:00 | 1 | 58362 | 1136 |
| 38 | KSC | 12:00 | 2 | 43306 | 925 |
| 39 | KSC | 12:00 | 3 | 30195 | 491 |
| 40 | KSC | 12:00 | 4 | 53258 | 901 |
| 41 | KSC | 12:00 | 5 | 29794 | 603 |
| 42 | KSC | 12:00 | 6 | 31039 | 624 |
| 43 | KSC | 18:00 | 1 | 40669 | 911 |
| 44 | KSC | 18:00 | 2 | 54562 | 937 |
| 45 | KSC | 18:00 | 3 | 42856 | 643 |
| 46 | KSC | 18:00 | 4 | 51580 | 875 |
| 47 | KSC | 18:00 | 5 | 34361 | 629 |
| 48 | KSC | 18:00 | 6 | 30277 | 617 |
| 49 | MON | 00:00 | 1 | 38537 | 606 |
| 50 | MON | 00:00 | 2 | 37697 | 723 |
| 51 | MON | 00:00 | 3 | 32671 | 568 |
| 52 | MON | 00:00 | 4 | 75073 | 1270 |
| 53 | MON | 00:00 | 5 | 52208 | 848 |
| 54 | MON | 00:00 | 6 | 66780 | 1109 |
| 55 | MON | 06:00 | 1 | 38358 | 689 |
| 56 | MON | 06:00 | 2 | 41303 | 731 |
| 57 | MON | 06:00 | 3 | 46495 | 898 |
| 58 | MON | 06:00 | 4 | 65807 | 869 |
| 59 | MON | 06:00 | 5 | 53688 | 850 |
| 60 | MON | 06:00 | 6 | 33389 | 516 |
| 61 | MON | 12:00 | 1 | 38091 | 689 |
| 62 | MON | 12:00 | 2 | 38143 | 627 |
| 63 | MON | 12:00 | 3 | 35048 | 492 |
| 64 | MON | 12:00 | 4 | 47537 | 597 |
| 65 | MON | 12:00 | 5 | 44979 | 635 |
| 66 | MON | 12:00 | 6 | 41379 | 831 |
| 67 | MON | 18:00 | 1 | 36989 | 568 |
| 68 | MON | 18:00 | 2 | 44345 | 827 |
| 69 | MON | 18:00 | 3 | 31040 | 360 |
| 70 | MON | 18:00 | 4 | 51538 | 820 |
| 71 | MON | 18:00 | 5 | 49378 | 688 |
| 72 | MON | 18:00 | 6 | 37258 | 667 |
| 73 | MOC | 00:00 | 1 | 47875 | 1183 |
| 74 | MOC | 00:00 | 2 | 34280 | 782 |
| 75 | MOC | 00:00 | 3 | 41693 | 751 |
| 76 | MOC | 00:00 | 4 | 63685 | 1109 |
| 77 | MOC | 00:00 | 5 | 61976 | 1115 |
| 78 | MOC | 00:00 | 6 | 31767 | 629 |
| 79 | MOC | 06:00 | 1 | 47875 | 1183 |
| 80 | MOC | 06:00 | 2 | 36218 | 806 |
| 81 | MOC | 06:00 | 3 | 41407 | 769 |
| 82 | MOC | 06:00 | 4 | 63717 | 1035 |
| 83 | MOC | 06:00 | 5 | 46648 | 841 |
| 84 | MOC | 06:00 | 6 | 42212 | 803 |
| 85 | MOC | 12:00 | 1 | 35561 | 742 |
| 86 | MOC | 12:00 | 2 | 43352 | 956 |
| 87 | MOC | 12:00 | 3 | 48467 | 773 |
| 88 | MOC | 12:00 | 4 | 58422 | 1018 |
| 89 | MOC | 12:00 | 5 | 46870 | 965 |
| 90 | MOC | 12:00 | 6 | 46821 | 847 |
| 91 | MOC | 18:00 | 1 | 36357 | 841 |
| 92 | MOC | 18:00 | 2 | 40310 | 765 |
| 93 | MOC | 18:00 | 3 | 58854 | 962 |
| 94 | MOC | 18:00 | 4 | 57592 | 1046 |
| 95 | MOC | 18:00 | 5 | 55608 | 1203 |
| 96 | MOC | 18:00 | 6 | 31587 | 737 |

**Table S2.** Network characteristics***** MO = Mark Oliphant CP, KS = Kenneth Stirling CP.

| **ID** | **Mean edge weight** | **Mean degree** | **Centralisation** | **Mean Distance** | **Edge density** |
| --- | --- | --- | --- | --- | --- |
| Phylum Cleared MO 1800 | 0.77 | 8.1 | 0.26 | 2.07 | 0.43 |
| Phylum Cleared MO 0000 | 0.69 | 2.8 | 0.11 | 3.20 | 0.15 |
| Phylum Cleared MO 0600 | 0.61 | 5.4 | 0.24 | 2.51 | 0.28 |
| Phylum Cleared MO 1200 | 0.79 | 6.0 | 0.26 | 2.44 | 0.32 |
| Phylum Cleared KS 1800 | 0.86 | 5.8 | 0.23 | 1.92 | 0.32 |
| Phylum Cleared KS 0000 | 0.76 | 5.3 | 0.26 | 2.40 | 0.24 |
| Phylum Cleared KS 0600 | 0.55 | 4.9 | 0.21 | 2.34 | 0.26 |
| Phylum Cleared KS 1200 | 0.79 | 7.0 | 0.22 | 1.94 | 0.32 |
| Phylum Native MO 1800 | 0.52 | 4.3 | 0.16 | 2.31 | 0.25 |
| Phylum Native MO 0000 | 0.88 | 6.9 | 0.27 | 1.99 | 0.36 |
| Phylum Native MO 0600 | 0.10 | 4.0 | 0.26 | 1.67 | 0.21 |
| Phylum Native MO 1200 | 0.36 | 5.0 | 0.23 | 1.83 | 0.38 |
| Phylum Native KS 1800 | 0.46 | 3.1 | 0.26 | 2.39 | 0.21 |
| Phylum Native KS 0000 | 0.68 | 3.9 | 0.23 | 2.77 | 0.18 |
| Phylum Native KS 0600 | 0.59 | 5.0 | 0.25 | 1.89 | 0.32 |
| Phylum Native KS 1200 | 0.88 | 3.7 | 0.23 | 2.03 | 0.19 |
| Genus Cleared MO 1800 | 0.80 | 5.0 | 0.85 | 4.99 | 0.02 |
| Genus Cleared MO 0000 | 0.89 | 4.6 | 0.04 | 2.75 | 0.02 |
| Genus Cleared MO 0600 | 0.71 | 4.7 | 0.12 | 1.60 | 0.02 |
| Genus Cleared MO 1200 | 0.50 | 4.5 | 0.07 | 3.62 | 0.02 |
| Genus Cleared KS 1800 | 0.78 | 3.7 | 0.05 | 1.44 | 0.02 |
| Genus Cleared KS 0000 | 0.88 | 3.79 | 0.03 | 1.26 | 0.02 |
| Genus Cleared KS 0600 | 0.85 | 4.71 | 0.04 | 1.87 | 0.02 |
| Genus Cleared KS 1200 | 0.75 | 5.3 | 0.05 | 3.35 | 0.02 |
| Genus Native MO 1800 | 0.92 | 4.18 | 0.67 | 1.24 | 0.02 |
| Genus Native MO 0000 | 0.94 | 4.59 | 0.06 | 1.87 | 0.02 |
| Genus Native MO 0600 | 0.78 | 3.26 | 0.06 | 1.57 | 0.02 |
| Genus Native MO 1200 | 0.69 | 3.45 | 0.06 | 1.99 | 0.02 |
| Genus Native KS 1800 | 0.87 | 3.80 | 0.05 | 2.04 | 0.02 |
| Genus Native KS 0000 | 0.78 | 4.35 | 0.04 | 1.51 | 0.02 |
| Genus Native KS 0600 | 0.92 | 6.05 | 0.05 | 1.66 | 0.03 |
| Genus Native KS 1200 | 0.97 | 1.2 | 0.20 | 1.25 | 0.30 |

* **Mean degree** = average number of interactions across the network

**Centralisation** = concentration or inequality of network connections among nodes

**Edge density** = how many connections are present out of all possible connections

**Table S3.** Cleared Kenneth Stirling – descriptive data for the 50 ASVs displaying the largest magnitude of log-fold change between comparison groups from ANCOM-BC differential abundance testing, compared to 00:00 hr baseline (as highlighted in Figure S2).

| Taxon | Comparison times | | Log-fold-change | Increasing or decreasing cf. 00:00 hr | Phylum | Class | Order | Family | Genus |
| --- | --- | --- | --- | --- | --- | --- | --- | --- | --- |
| ASV_240 | 18:00 | -3.13 | | Decreasing | Proteobacteria | Gammaproteobacteria | Burkholderiales | Unclassified | Unclassified |
| ASV_1019 | 12:00 | -2.97 | | Decreasing | Actinobacteriota | Acidimicrobiia | IMCC26256 | IMCC26256 | IMCC26256 |
| ASV_1432 | 18:00 | -2.95 | | Decreasing | Proteobacteria | Alphaproteobacteria | Rhizobiales | Xanthobacteraceae | uncultured |
| ASV_1008 | 12:00 | -2.92 | | Decreasing | Actinobacteriota | Thermoleophilia | Solirubrobacterales | Solirubrobacteraceae | Conexibacter |
| ASV_1282 | 06:00 | -2.85 | | Decreasing | Actinobacteriota | Thermoleophilia | Solirubrobacterales | Solirubrobacteraceae | Conexibacter |
| ASV_1276 | 06:00 | -2.80 | | Decreasing | Actinobacteriota | Actinobacteria | Streptomycetales | Streptomycetaceae | Streptomyces |
| ASV_1858 | 06:00 | -2.75 | | Decreasing | Chloroflexi | Ktedonobacteria | B12-WMSP1 | B12-WMSP1 | B12-WMSP1 |
| ASV_1009 | 06:00 | -2.72 | | Decreasing | Actinobacteriota | Thermoleophilia | Solirubrobacterales | Solirubrobacteraceae | Solirubrobacter |
| ASV_2105 | 06:00 | -2.65 | | Decreasing | Actinobacteriota | Thermoleophilia | Gaiellales | uncultured | uncultured |
| ASV_1478 | 06:00 | -2.65 | | Decreasing | Actinobacteriota | Actinobacteria | Frankiales | Unclassified | Unclassified |
| ASV_1009 | 18:00 | -2.63 | | Decreasing | Actinobacteriota | Thermoleophilia | Solirubrobacterales | Solirubrobacteraceae | Solirubrobacter |
| ASV_305 | 06:00 | -2.57 | | Decreasing | Acidobacteriota | Acidobacteriae | Acidobacteriales | Acidobacteriaceae | Edaphobacter |
| ASV_1214 | 18:00 | -2.57 | | Decreasing | Acidobacteriota | Acidobacteriae | Solibacterales | Solibacteraceae | Candidatus_Solibacter |
| ASV_663 | 12:00 | -2.54 | | Decreasing | Actinobacteriota | Actinobacteria | Kineosporiales | Kineosporiaceae | Unclassified |
| ASV_663 | 06:00 | -2.54 | | Decreasing | Actinobacteriota | Actinobacteria | Kineosporiales | Kineosporiaceae | Unclassified |
| ASV_70 | 12:00 | -2.51 | | Decreasing | Proteobacteria | Alphaproteobacteria | Rhizobiales | Xanthobacteraceae | Unclassified |
| ASV_1555 | 06:00 | -2.47 | | Decreasing | Chloroflexi | Ktedonobacteria | B12-WMSP1 | B12-WMSP1 | B12-WMSP1 |
| ASV_2761 | 18:00 | -2.45 | | Decreasing | Actinobacteriota | Actinobacteria | Propionibacteriales | Nocardioidaceae | Kribbella |
| ASV_1925 | 06:00 | -2.45 | | Decreasing | Actinobacteriota | Actinobacteria | Unclassified | Unclassified | Unclassified |
| ASV_2761 | 06:00 | -2.44 | | Decreasing | Actinobacteriota | Actinobacteria | Propionibacteriales | Nocardioidaceae | Kribbella |
| ASV_1592 | 12:00 | -2.43 | | Decreasing | Proteobacteria | Alphaproteobacteria | Caulobacterales | Caulobacteraceae | uncultured |
| ASV_1941 | 06:00 | 2.44 | | Increasing | Proteobacteria | Alphaproteobacteria | Micropepsales | Micropepsaceae | uncultured |
| ASV_1456 | 12:00 | 2.44 | | Increasing | Actinobacteriota | Actinobacteria | Propionibacteriales | Nocardioidaceae | Nocardioides |
| ASV_1278 | 06:00 | 2.44 | | Increasing | Actinobacteriota | Actinobacteria | Frankiales | Frankiaceae | Jatrophihabitans |
| ASV_1136 | 06:00 | 2.45 | | Increasing | Actinobacteriota | Thermoleophilia | Gaiellales | uncultured | uncultured |
| ASV_2287 | 18:00 | 2.46 | | Increasing | Actinobacteriota | Thermoleophilia | Unclassified | Unclassified | Unclassified |
| ASV_1638 | 12:00 | 2.47 | | Increasing | Actinobacteriota | Actinobacteria | Catenulisporales | Catenulisporaceae | Catenulispora |
| ASV_1485 | 06:00 | 2.52 | | Increasing | Proteobacteria | Alphaproteobacteria | Dongiales | Dongiaceae | Dongia |
| ASV_167 | 18:00 | 2.52 | | Increasing | Actinobacteriota | Actinobacteria | Frankiales | Frankiaceae | Jatrophihabitans |
| ASV_2541 | 18:00 | 2.52 | | Increasing | Proteobacteria | Alphaproteobacteria | Rhizobiales | Xanthobacteraceae | uncultured |
| ASV_3151 | 12:00 | 2.53 | | Increasing | Actinobacteriota | Thermoleophilia | Gaiellales | uncultured | uncultured |
| ASV_805 | 06:00 | 2.56 | | Increasing | Acidobacteriota | Acidobacteriae | Acidobacteriales | uncultured | uncultured |
| ASV_50 | 18:00 | 2.58 | | Increasing | Actinobacteriota | Thermoleophilia | Solirubrobacterales | Solirubrobacteraceae | Conexibacter |
| ASV_1426 | 06:00 | 2.58 | | Increasing | Proteobacteria | Alphaproteobacteria | Micropepsales | Micropepsaceae | uncultured |
| ASV_3501 | 18:00 | 2.63 | | Increasing | Actinobacteriota | Actinobacteria | Streptomycetales | Streptomycetaceae | Streptomyces |
| ASV_1562 | 18:00 | 2.69 | | Increasing | Actinobacteriota | Thermoleophilia | Solirubrobacterales | Unclassified | Unclassified |
| ASV_180 | 06:00 | 2.70 | | Increasing | Acidobacteriota | Acidobacteriae | Acidobacteriales | Koribacteraceae | Candidatus_Koribacter |
| ASV_1204 | 12:00 | 2.74 | | Increasing | Actinobacteriota | Thermoleophilia | Gaiellales | Gaiellaceae | Gaiella |
| ASV_1562 | 06:00 | 2.75 | | Increasing | Actinobacteriota | Thermoleophilia | Solirubrobacterales | Unclassified | Unclassified |
| ASV_244 | 12:00 | 2.75 | | Increasing | Proteobacteria | Alphaproteobacteria | Rhizobiales | Xanthobacteraceae | uncultured |
| ASV_53 | 18:00 | 2.78 | | Increasing | Actinobacteriota | Thermoleophilia | Solirubrobacterales | Solirubrobacteraceae | Conexibacter |
| ASV_1000 | 18:00 | 2.80 | | Increasing | Actinobacteriota | Thermoleophilia | Gaiellales | uncultured | uncultured |
| ASV_540 | 18:00 | 2.82 | | Increasing | Myxococcota | Polyangia | Haliangiales | Haliangiaceae | Haliangium |
| ASV_85 | 06:00 | 2.83 | | Increasing | Proteobacteria | Alphaproteobacteria | Micropepsales | Micropepsaceae | uncultured |
| ASV_159 | 18:00 | 2.85 | | Increasing | Proteobacteria | Alphaproteobacteria | Rhizobiales | KF-JG30-B3 | KF-JG30-B3 |
| ASV_255 | 18:00 | 3.10 | | Increasing | Actinobacteriota | MB-A2-108 | MB-A2-108 | MB-A2-108 | MB-A2-108 |
| ASV_552 | 06:00 | 3.12 | | Increasing | Proteobacteria | Alphaproteobacteria | Rhizobiales | Xanthobacteraceae | uncultured |
| ASV_381 | 18:00 | 3.15 | | Increasing | Actinobacteriota | Thermoleophilia | Solirubrobacterales | 67-14 | 67-14 |
| ASV_552 | 12:00 | 3.18 | | Increasing | Proteobacteria | Alphaproteobacteria | Rhizobiales | Xanthobacteraceae | uncultured |
| ASV_1512 | 12:00 | 3.21 | | Increasing | Chloroflexi | KD4-96 | KD4-96 | KD4-96 | KD4-96 |
| ASV_1290 | 18:00 | 3.27 | | Increasing | Actinobacteriota | Actinobacteria | Streptomycetales | Streptomycetaceae | Streptomyces |
| ASV_755 | 18:00 | 3.29 | | Increasing | Acidobacteriota | Acidobacteriae | Bryobacterales | Bryobacteraceae | Bryobacter |
| ASV_741 | 18:00 | 3.39 | | Increasing | Actinobacteriota | Thermoleophilia | Gaiellales | uncultured | uncultured |
| ASV_1878 | 12:00 | 3.43 | | Increasing | Actinobacteriota | Thermoleophilia | Solirubrobacterales | 67-14 | 67-14 |
| ASV_1205 | 06:00 | 3.51 | | Increasing | Actinobacteriota | Thermoleophilia | Solirubrobacterales | 67-14 | 67-14 |

**Table S4.** Native Kenneth Stirling – descriptive data for the 50 ASVs displaying the largest magnitude of log-fold change between comparison groups from ANCOM-BC differential abundance testing, compared to 00:00 hr baseline (as highlighted in Figure S3).

| Taxon | Comparison time | | Log-fold-change | | Increasing or decreasing cf. 00:00 hr | Phylum | Class | Order | Family | | Genus |
| --- | --- | --- | --- | --- | --- | --- | --- | --- | --- | --- | --- |
| ASV_36 | | 06:00 | | -3.49 | Decreasing | Proteobacteria | Gammaproteobacteria | Burkholderiales | | Oxalobacteraceae | Unclassified |
| ASV_189 | | 12:00 | | -3.08 | Decreasing | Acidobacteriota | Acidobacteriae | Acidobacteriales | | Koribacteraceae | Candidatus_Koribacter |
| ASV_844 | | 06:00 | | -3.06 | Decreasing | Proteobacteria | Gammaproteobacteria | Gammaproteobacteria_Incertae_Sedis | | Unknown_Family | Acidibacter |
| ASV_193 | | 06:00 | | -2.93 | Decreasing | Acidobacteriota | Acidobacteriae | Acidobacteriales | | Acidobacteriaceae_(Subgroup_1) | Granulicella |
| ASV_172 | | 12:00 | | -2.92 | Decreasing | Proteobacteria | Alphaproteobacteria | Rhizobiales | | Xanthobacteraceae | uncultured |
| ASV_97 | | 18:00 | | -2.92 | Decreasing | Proteobacteria | Alphaproteobacteria | Caulobacterales | | Caulobacteraceae | Phenylobacterium |
| ASV_141 | | 06:00 | | -2.89 | Decreasing | Acidobacteriota | Acidobacteriae | Acidobacteriales | | Acidobacteriaceae_(Subgroup_1) | Occallatibacter |
| ASV_317 | | 12:00 | | -2.82 | Decreasing | Actinobacteriota | Acidimicrobiia | uncultured | | uncultured | uncultured |
| ASV_70 | | 06:00 | | -2.79 | Decreasing | Proteobacteria | Alphaproteobacteria | Rhizobiales | | Xanthobacteraceae | Unclassified |
| ASV_725 | | 06:00 | | -2.75 | Decreasing | Acidobacteriota | Acidobacteriae | Subgroup_2 | | Subgroup_2 | Subgroup_2 |
| ASV_257 | | 06:00 | | -2.72 | Decreasing | Acidobacteriota | Acidobacteriae | Acidobacteriales | | Acidobacteriaceae_(Subgroup_1) | Granulicella |
| ASV_433 | | 06:00 | | -2.70 | Decreasing | Actinobacteriota | Actinobacteria | Frankiales | | Acidothermaceae | Acidothermus |
| ASV_391 | | 12:00 | | -2.69 | Decreasing | Proteobacteria | Alphaproteobacteria | Caulobacterales | | Caulobacteraceae | Unclassified |
| ASV_1662 | | 18:00 | | -2.65 | Decreasing | Actinobacteriota | Thermoleophilia | Gaiellales | | Unclassified | Unclassified |
| ASV_391 | | 18:00 | | -2.62 | Decreasing | Proteobacteria | Alphaproteobacteria | Caulobacterales | | Caulobacteraceae | Unclassified |
| ASV_766 | | 12:00 | | -2.61 | Decreasing | Proteobacteria | Alphaproteobacteria | Rhizobiales | | Xanthobacteraceae | uncultured |
| ASV_329 | | 06:00 | | -2.56 | Decreasing | Proteobacteria | Gammaproteobacteria | Burkholderiales | | SC-I-84 | SC-I-84 |
| ASV_266 | | 06:00 | | -2.56 | Decreasing | Actinobacteriota | Thermoleophilia | Solirubrobacterales | | Solirubrobacteraceae | Conexibacter |
| ASV_2029 | | 12:00 | | -2.53 | Decreasing | Acidobacteriota | Acidobacteriae | Acidobacteriales | | uncultured | uncultured |
| ASV_766 | | 06:00 | | -2.52 | Decreasing | Proteobacteria | Alphaproteobacteria | Rhizobiales | | Xanthobacteraceae | uncultured |
| ASV_47 | | 06:00 | | -2.48 | Decreasing | Proteobacteria | Alphaproteobacteria | Rhizobiales | | Xanthobacteraceae | Pseudolabrys |
| ASV_517 | | 12:00 | | -2.46 | Decreasing | Acidobacteriota | Blastocatellia | Elev-16S-573 | | Elev-16S-573 | Elev-16S-573 |
| ASV_111 | | 06:00 | | -2.45 | Decreasing | Acidobacteriota | Acidobacteriae | Acidobacteriales | | uncultured | uncultured |
| ASV_2654 | | 12:00 | | -2.41 | Decreasing | Proteobacteria | Alphaproteobacteria | Acetobacterales | | Acetobacteraceae | Acidiphilium |
| ASV_2029 | | 06:00 | | -2.41 | Decreasing | Acidobacteriota | Acidobacteriae | Acidobacteriales | | uncultured | uncultured |
| ASV_202 | | 06:00 | | -2.37 | Decreasing | Actinobacteriota | Thermoleophilia | Solirubrobacterales | | Solirubrobacteraceae | Conexibacter |
| ASV_878 | | 18:00 | | -2.34 | Decreasing | Actinobacteriota | Thermoleophilia | Solirubrobacterales | | 67-14 | 67-14 |
| ASV_2654 | | 18:00 | | -2.34 | Decreasing | Proteobacteria | Alphaproteobacteria | Acetobacterales | | Acetobacteraceae | Acidiphilium |
| ASV_2654 | | 06:00 | | -2.32 | Decreasing | Proteobacteria | Alphaproteobacteria | Acetobacterales | | Acetobacteraceae | Acidiphilium |
| ASV_664 | | 06:00 | | -2.29 | Decreasing | Acidobacteriota | Acidobacteriae | Acidobacteriales | | Acidobacteriaceae_(Subgroup_1) | Granulicella |
| ASV_2316 | | 12:00 | | -2.28 | Decreasing | Proteobacteria | Gammaproteobacteria | Burkholderiales | | Comamonadaceae | Rhizobacter |
| ASV_2501 | | 06:00 | | -2.27 | Decreasing | Chloroflexi | KD4-96 | KD4-96 | | KD4-96 | KD4-96 |
| ASV_710 | | 06:00 | | -2.26 | Decreasing | Acidobacteriota | Acidobacteriae | Subgroup_2 | | Subgroup_2 | Subgroup_2 |
| ASV_37 | | 06:00 | | -2.25 | Decreasing | Acidobacteriota | Acidobacteriae | Acidobacteriales | | uncultured | uncultured |
| ASV_37 | | 12:00 | | -2.23 | Decreasing | Acidobacteriota | Acidobacteriae | Acidobacteriales | | uncultured | uncultured |
| ASV_1728 | | 18:00 | | -2.21 | Decreasing | Actinobacteriota | Thermoleophilia | Solirubrobacterales | | Solirubrobacteraceae | Conexibacter |
| ASV_218 | | 06:00 | | -2.20 | Decreasing | Proteobacteria | Alphaproteobacteria | Rhizobiales | | Xanthobacteraceae | Unclassified |
| ASV_63 | | 06:00 | | -2.19 | Decreasing | Acidobacteriota | Acidobacteriae | Subgroup_2 | | Subgroup_2 | Subgroup_2 |
| ASV_443 | | 18:00 | | 2.22 | Increasing | Acidobacteriota | Blastocatellia | Elev-16S-573 | | Elev-16S-573 | Elev-16S-573 |
| ASV_181 | | 18:00 | | 2.23 | Increasing | Proteobacteria | Alphaproteobacteria | Micropepsales | | Micropepsaceae | uncultured |
| ASV_866 | | 12:00 | | 2.25 | Increasing | Proteobacteria | Gammaproteobacteria | Pseudomonadales | | Pseudomonadaceae | Pseudomonas |
| ASV_1069 | | 18:00 | | 2.26 | Increasing | Actinobacteriota | Thermoleophilia | Solirubrobacterales | | 67-14 | 67-14 |
| ASV_1827 | | 18:00 | | 2.26 | Increasing | Acidobacteriota | Acidobacteriae | Unclassified | | Unclassified | Unclassified |
| ASV_2943 | | 18:00 | | 2.26 | Increasing | Acidobacteriota | Acidobacteriae | Solibacterales | | Solibacteraceae | Candidatus_Solibacter |
| ASV_294 | | 18:00 | | 2.27 | Increasing | Proteobacteria | Alphaproteobacteria | Caulobacterales | | Caulobacteraceae | Phenylobacterium |
| ASV_127 | | 06:00 | | 2.30 | Increasing | Actinobacteriota | Actinobacteria | Frankiales | | Acidothermaceae | Acidothermus |
| ASV_2175 | | 12:00 | | 2.30 | Increasing | Proteobacteria | Alphaproteobacteria | Elsterales | | uncultured | uncultured |
| ASV_563 | | 12:00 | | 2.34 | Increasing | Actinobacteriota | Thermoleophilia | Solirubrobacterales | | Solirubrobacteraceae | Conexibacter |
| ASV_65 | | 18:00 | | 2.39 | Increasing | Actinobacteriota | Thermoleophilia | Solirubrobacterales | | Solirubrobacteraceae | Conexibacter |
| ASV_378 | | 12:00 | | 2.43 | Increasing | Proteobacteria | Gammaproteobacteria | Xanthomonadales | | Rhodanobacteraceae | Rhodanobacter |
| ASV_626 | | 06:00 | | 2.44 | Increasing | Actinobacteriota | Thermoleophilia | Solirubrobacterales | | Solirubrobacteraceae | Conexibacter |
| ASV_711 | | 12:00 | | 2.46 | Increasing | Actinobacteriota | Thermoleophilia | Solirubrobacterales | | Solirubrobacteraceae | Conexibacter |
| ASV_583 | | 12:00 | | 2.75 | Increasing | Actinobacteriota | Acidimicrobiia | uncultured | | uncultured | uncultured |
| ASV_763 | | 12:00 | | 2.91 | Increasing | Actinobacteriota | Thermoleophilia | Gaiellales | | uncultured | uncultured |
| ASV_320 | | 18:00 | | 2.95 | Increasing | Actinobacteriota | Thermoleophilia | Solirubrobacterales | | Solirubrobacteraceae | Conexibacter |
| ASV_533 | | 12:00 | | 3.42 | Increasing | Proteobacteria | Alphaproteobacteria | Elsterales | | uncultured | uncultured |

**Table S5.** Cleared Mark Oliphant – descriptive data for the 50 ASVs displaying the largest magnitude of log-fold change between comparison groups from ANCOM-BC differential abundance testing, compared to 00:00 hr baseline (as highlighted in Figure S4).

| Taxon | Comparison times | Log-fold-change | Increasing or decreasing cf. 00:00 hr | Phylum | Class | Order | Family | Genus |
| --- | --- | --- | --- | --- | --- | --- | --- | --- |
| ASV_362 | 06:00 | -3.58 | Decreasing | Proteobacteria | Alphaproteobacteria | Rhizobiales | Xanthobacteraceae | uncultured |
| ASV_436 | 12:00 | -3.00 | Decreasing | Actinobacteriota | Actinobacteria | Corynebacteriales | Mycobacteriaceae | Mycobacterium |
| ASV_751 | 18:00 | -2.94 | Decreasing | Actinobacteriota | Thermoleophilia | Solirubrobacterales | Solirubrobacteraceae | uncultured |
| ASV_59 | 06:00 | -2.77 | Decreasing | Proteobacteria | Alphaproteobacteria | Rhizobiales | Beijerinckiaceae | Roseiarcus |
| ASV_113 | 18:00 | -2.73 | Decreasing | Proteobacteria | Alphaproteobacteria | Rhizobiales | Xanthobacteraceae | uncultured |
| ASV_2006 | 12:00 | -2.73 | Decreasing | Actinobacteriota | Actinobacteria | Frankiales | uncultured | uncultured |
| ASV_1264 | 18:00 | -2.68 | Decreasing | Acidobacteriota | Acidobacteriae | Bryobacterales | Bryobacteraceae | Bryobacter |
| ASV_1192 | 18:00 | -2.68 | Decreasing | Proteobacteria | Alphaproteobacteria | Rhizobiales | Beijerinckiaceae | Beijerinckiaceae |
| ASV_362 | 18:00 | -2.66 | Decreasing | Proteobacteria | Alphaproteobacteria | Rhizobiales | Xanthobacteraceae | uncultured |
| ASV_2235 | 12:00 | -2.62 | Decreasing | Acidobacteriota | Acidobacteriae | Subgroup_2 | Subgroup_2 | Subgroup_2 |
| ASV_362 | 12:00 | -2.62 | Decreasing | Proteobacteria | Alphaproteobacteria | Rhizobiales | Xanthobacteraceae | uncultured |
| ASV_487 | 12:00 | -2.61 | Decreasing | Proteobacteria | Alphaproteobacteria | Rhizobiales | Xanthobacteraceae | Pseudolabrys |
| ASV_987 | 12:00 | -2.56 | Decreasing | Actinobacteriota | Thermoleophilia | Solirubrobacterales | Solirubrobacteraceae | Conexibacter |
| ASV_1400 | 18:00 | -2.55 | Decreasing | Proteobacteria | Alphaproteobacteria | Rhizobiales | Beijerinckiaceae | Beijerinckiaceae |
| ASV_987 | 18:00 | -2.55 | Decreasing | Actinobacteriota | Thermoleophilia | Solirubrobacterales | Solirubrobacteraceae | Conexibacter |
| ASV_562 | 18:00 | -2.51 | Decreasing | Actinobacteriota | Thermoleophilia | Gaiellales | uncultured | uncultured |
| ASV_4021 | 12:00 | -2.50 | Decreasing | Chloroflexi | AD3 | AD3 | AD3 | AD3 |
| ASV_4021 | 18:00 | -2.49 | Decreasing | Chloroflexi | AD3 | AD3 | AD3 | AD3 |
| ASV_744 | 06:00 | -2.48 | Decreasing | Actinobacteriota | Thermoleophilia | Solirubrobacterales | Unclassified | Unclassified |
| ASV_78 | 12:00 | -2.36 | Decreasing | Proteobacteria | Alphaproteobacteria | Rhizobiales | Xanthobacteraceae | Unclassified |
| ASV_2743 | 12:00 | -2.36 | Decreasing | Proteobacteria | Alphaproteobacteria | Rhodospirillales | Magnetospirillaceae | uncultured |
| ASV_239 | 12:00 | -2.35 | Decreasing | Patescibacteria | Saccharimonadia | Saccharimonadales | WWH38 | WWH38 |
| ASV_1834 | 06:00 | -2.33 | Decreasing | Actinobacteriota | Thermoleophilia | Solirubrobacterales | Unclassified | Unclassified |
| ASV_2281 | 18:00 | -2.30 | Decreasing | Actinobacteriota | Actinobacteria | Corynebacteriales | Mycobacteriaceae | Mycobacterium |
| ASV_723 | 18:00 | -2.30 | Decreasing | Acidobacteriota | Acidobacteriae | Acidobacteriales | uncultured | uncultured |
| ASV_166 | 06:00 | -2.30 | Decreasing | Proteobacteria | Alphaproteobacteria | Rhizobiales | Xanthobacteraceae | Bradyrhizobium |
| ASV_278 | 06:00 | 2.25 | Increasing | Proteobacteria | Alphaproteobacteria | Reyranellales | Reyranellaceae | Reyranella |
| ASV_4170 | 18:00 | 2.25 | Increasing | Chloroflexi | Ktedonobacteria | Ktedonobacterales | Ktedonobacteraceae | Unclassified |
| ASV_795 | 06:00 | 2.29 | Increasing | Actinobacteriota | Actinobacteria | Streptomycetales | Streptomycetaceae | Streptomyces |
| ASV_3881 | 12:00 | 2.35 | Increasing | Actinobacteriota | Thermoleophilia | Gaiellales | uncultured | uncultured |
| ASV_1903 | 18:00 | 2.40 | Increasing | Gemmatimonadota | Gemmatimonadetes | Gemmatimonadales | Gemmatimonadaceae | uncultured |
| ASV_1724 | 06:00 | 2.42 | Increasing | Chloroflexi | KD4-96 | KD4-96 | KD4-96 | KD4-96 |
| ASV_2286 | 12:00 | 2.48 | Increasing | Proteobacteria | Alphaproteobacteria | uncultured | uncultured | uncultured |
| ASV_376 | 18:00 | 2.49 | Increasing | Proteobacteria | Gammaproteobacteria | Burkholderiales | SC-I-84 | SC-I-84 |
| ASV_971 | 18:00 | 2.51 | Increasing | Actinobacteriota | Actinobacteria | Corynebacteriales | Mycobacteriaceae | Mycobacterium |
| ASV_783 | 06:00 | 2.54 | Increasing | Proteobacteria | Alphaproteobacteria | Rhizobiales | Xanthobacteraceae | uncultured |
| ASV_30 | 06:00 | 2.56 | Increasing | Proteobacteria | Alphaproteobacteria | Rhizobiales | Beijerinckiaceae | Roseiarcus |
| ASV_746 | 12:00 | 2.57 | Increasing | Actinobacteriota | Actinobacteria | Micrococcales | Intrasporangiaceae | Unclassified |
| ASV_467 | 12:00 | 2.57 | Increasing | Actinobacteriota | Thermoleophilia | Solirubrobacterales | Unclassified | Unclassified |
| ASV_1888 | 18:00 | 2.58 | Increasing | Myxococcota | Polyangia | Haliangiales | Haliangiaceae | Haliangium |
| ASV_3962 | 18:00 | 2.63 | Increasing | Acidobacteriota | Acidobacteriae | Acidobacteriales | uncultured | uncultured |
| ASV_1315 | 06:00 | 2.63 | Increasing | Chloroflexi | Ktedonobacteria | B12-WMSP1 | B12-WMSP1 | B12-WMSP1 |
| ASV_157 | 12:00 | 2.65 | Increasing | Actinobacteriota | Thermoleophilia | Solirubrobacterales | Solirubrobacteraceae | Conexibacter |
| ASV_50 | 12:00 | 2.74 | Increasing | Actinobacteriota | Thermoleophilia | Solirubrobacterales | Solirubrobacteraceae | Conexibacter |
| ASV_971 | 12:00 | 2.75 | Increasing | Actinobacteriota | Actinobacteria | Corynebacteriales | Mycobacteriaceae | Mycobacterium |
| ASV_3406 | 18:00 | 2.75 | Increasing | Proteobacteria | Alphaproteobacteria | Micropepsales | Micropepsaceae | uncultured |
| ASV_674 | 18:00 | 2.80 | Increasing | Acidobacteriota | Acidobacteriae | Bryobacterales | Bryobacteraceae | Bryobacter |
| ASV_601 | 12:00 | 2.80 | Increasing | Acidobacteriota | Acidobacteriae | Acidobacteriales | uncultured | uncultured |
| ASV_1324 | 12:00 | 2.82 | Increasing | Actinobacteriota | Thermoleophilia | Solirubrobacterales | Unclassified | Unclassified |
| ASV_1119 | 18:00 | 2.89 | Increasing | Acidobacteriota | Acidobacteriae | Bryobacterales | Bryobacteraceae | Bryobacter |
| ASV_793 | 12:00 | 2.99 | Increasing | Actinobacteriota | Thermoleophilia | Gaiellales | uncultured | uncultured |
| ASV_86 | 12:00 | 3.06 | Increasing | Actinobacteriota | Thermoleophilia | Solirubrobacterales | Solirubrobacteraceae | Conexibacter |
| ASV_894 | 12:00 | 3.07 | Increasing | Actinobacteriota | Thermoleophilia | Solirubrobacterales | Solirubrobacteraceae | Conexibacter |
| ASV_1816 | 12:00 | 3.10 | Increasing | Actinobacteriota | Actinobacteria | Frankiales | Acidothermaceae | Acidothermus |
| ASV_86 | 06:00 | 3.15 | Increasing | Actinobacteriota | Thermoleophilia | Solirubrobacterales | Solirubrobacteraceae | Conexibacter |
| ASV_2513 | 12:00 | 3.30 | Increasing | WPS-2 | WPS-2 | WPS-2 | WPS-2 | WPS-2 |

**Table S6.** Native Mark Oliphant – descriptive data for the 50 ASVs displaying the largest magnitude of log-fold change between comparison groups from ANCOM-BC differential abundance testing, compared to 00:00 hr baseline (as highlighted in Figure S5).

| Taxon | Comparison times | Log-fold-change | Increasing or decreasing cf. 00:00 hr | Phylum | Class | Order | Family | Genus |
| --- | --- | --- | --- | --- | --- | --- | --- | --- |
| ASV_48 | 18:00 | -3.90 | Decreasing | Proteobacteria | Alphaproteobacteria | Acetobacterales | Acetobacteraceae | Acidicaldus |
| ASV_1053 | 06:00 | -3.75 | Decreasing | Proteobacteria | Alphaproteobacteria | Acetobacterales | Acetobacteraceae | Acidocella |
| ASV_1053 | 18:00 | -3.73 | Decreasing | Proteobacteria | Alphaproteobacteria | Acetobacterales | Acetobacteraceae | Acidocella |
| ASV_128 | 18:00 | -3.64 | Decreasing | Actinobacteriota | Thermoleophilia | Gaiellales | uncultured | uncultured |
| ASV_131 | 06:00 | -3.50 | Decreasing | Proteobacteria | Alphaproteobacteria | Caulobacterales | Caulobacteraceae | Caulobacter |
| ASV_131 | 12:00 | -3.49 | Decreasing | Proteobacteria | Alphaproteobacteria | Caulobacterales | Caulobacteraceae | Caulobacter |
| ASV_1051 | 12:00 | -3.30 | Decreasing | Proteobacteria | Alphaproteobacteria | Rhizobiales | Xanthobacteraceae | Afipia |
| ASV_558 | 06:00 | -3.30 | Decreasing | Proteobacteria | Alphaproteobacteria | Caulobacterales | Caulobacteraceae | Unclassified |
| ASV_1441 | 06:00 | -3.22 | Decreasing | Proteobacteria | Gammaproteobacteria | Xanthomonadales | Rhodanobacteraceae | Rhodanobacter |
| ASV_2324 | 12:00 | -3.22 | Decreasing | Proteobacteria | Gammaproteobacteria | Burkholderiales | Burkholderiaceae | Burkholderia-Caballeronia-Paraburkholderia |
| ASV_2324 | 18:00 | -3.22 | Decreasing | Proteobacteria | Gammaproteobacteria | Burkholderiales | Burkholderiaceae | Burkholderia-Caballeronia-Paraburkholderia |
| ASV_312 | 18:00 | -3.21 | Decreasing | Proteobacteria | Gammaproteobacteria | Burkholderiales | Burkholderiaceae | Burkholderia-Caballeronia-Paraburkholderia |
| ASV_872 | 18:00 | -3.09 | Decreasing | Proteobacteria | Alphaproteobacteria | Rhodospirillales | Rhodospirillaceae | uncultured |
| ASV_871 | 06:00 | -3.07 | Decreasing | Acidobacteriota | Acidobacteriae | Acidobacteriales | Koribacteraceae | Candidatus_Koribacter |
| ASV_648 | 06:00 | -3.05 | Decreasing | Proteobacteria | Gammaproteobacteria | JG36-TzT-191 | JG36-TzT-191 | JG36-TzT-191 |
| ASV_871 | 18:00 | -3.05 | Decreasing | Acidobacteriota | Acidobacteriae | Acidobacteriales | Koribacteraceae | Candidatus_Koribacter |
| ASV_47 | 12:00 | -3.04 | Decreasing | Proteobacteria | Alphaproteobacteria | Rhizobiales | Xanthobacteraceae | Pseudolabrys |
| ASV_140 | 12:00 | -3.02 | Decreasing | Proteobacteria | Alphaproteobacteria | Elsterales | uncultured | uncultured |
| ASV_523 | 06:00 | -3.01 | Decreasing | Acidobacteriota | Acidobacteriae | Acidobacteriales | Acidobacteriaceae_(Subgroup_1) | Granulicella |
| ASV_1949 | 12:00 | -3.00 | Decreasing | Proteobacteria | Alphaproteobacteria | uncultured | uncultured | uncultured |
| ASV_128 | 12:00 | -2.99 | Decreasing | Actinobacteriota | Thermoleophilia | Gaiellales | uncultured | uncultured |
| ASV_462 | 18:00 | -2.99 | Decreasing | Acidobacteriota | Acidobacteriae | Acidobacteriales | uncultured | uncultured |
| ASV_704 | 06:00 | -2.94 | Decreasing | Proteobacteria | Alphaproteobacteria | Caulobacterales | Caulobacteraceae | Brevundimonas |
| ASV_382 | 12:00 | -2.93 | Decreasing | Acidobacteriota | Acidobacteriae | Acidobacteriales | Acidobacteriaceae_(Subgroup_1) | Edaphobacter |
| ASV_974 | 06:00 | -2.92 | Decreasing | Proteobacteria | Alphaproteobacteria | Elsterales | URHD0088 | URHD0088 |
| ASV_1257 | 18:00 | -2.91 | Decreasing | Acidobacteriota | Acidobacteriae | Acidobacteriales | Acidobacteriaceae_(Subgroup_1) | Acidipila-Silvibacterium |
| ASV_71 | 12:00 | -2.91 | Decreasing | Proteobacteria | Gammaproteobacteria | Burkholderiales | Nitrosomonadaceae | Ellin6067 |
| ASV_615 | 12:00 | -2.88 | Decreasing | Proteobacteria | Gammaproteobacteria | WD260 | WD260 | WD260 |
| ASV_525 | 18:00 | -2.87 | Decreasing | Acidobacteriota | Acidobacteriae | Subgroup_2 | Subgroup_2 | Subgroup_2 |
| ASV_1413 | 06:00 | -2.86 | Decreasing | Acidobacteriota | Acidobacteriae | Acidobacteriales | Acidobacteriaceae_(Subgroup_1) | Unclassified |
| ASV_2663 | 06:00 | -2.84 | Decreasing | Acidobacteriota | Acidobacteriae | Acidobacteriales | uncultured | uncultured |
| ASV_355 | 12:00 | -2.82 | Decreasing | Acidobacteriota | Acidobacteriae | Acidobacteriales | uncultured | uncultured |
| ASV_2663 | 12:00 | -2.82 | Decreasing | Acidobacteriota | Acidobacteriae | Acidobacteriales | uncultured | uncultured |
| ASV_2663 | 18:00 | -2.81 | Decreasing | Acidobacteriota | Acidobacteriae | Acidobacteriales | uncultured | uncultured |
| ASV_2031 | 12:00 | -2.80 | Decreasing | Proteobacteria | Gammaproteobacteria | WD260 | WD260 | WD260 |
| ASV_294 | 12:00 | -2.80 | Decreasing | Proteobacteria | Alphaproteobacteria | Caulobacterales | Caulobacteraceae | Phenylobacterium |
| ASV_1619 | 12:00 | -2.80 | Decreasing | Proteobacteria | Gammaproteobacteria | Gammaproteobacteria_Incertae_Sedis | Unknown_Family | Acidibacter |
| ASV_2031 | 18:00 | -2.80 | Decreasing | Proteobacteria | Gammaproteobacteria | WD260 | WD260 | WD260 |
| ASV_1313 | 12:00 | -2.79 | Decreasing | Acidobacteriota | Acidobacteriae | Subgroup_2 | Subgroup_2 | Subgroup_2 |
| ASV_1604 | 18:00 | -2.78 | Decreasing | Proteobacteria | Alphaproteobacteria | Elsterales | Elsteraceae | uncultured |
| ASV_2326 | 06:00 | -2.78 | Decreasing | Acidobacteriota | Acidobacteriae | Acidobacteriales | Acidobacteriaceae_(Subgroup_1) | Occallatibacter |
| ASV_1413 | 18:00 | -2.76 | Decreasing | Acidobacteriota | Acidobacteriae | Acidobacteriales | Acidobacteriaceae_(Subgroup_1) | Unclassified |
| ASV_2326 | 18:00 | -2.76 | Decreasing | Acidobacteriota | Acidobacteriae | Acidobacteriales | Acidobacteriaceae_(Subgroup_1) | Occallatibacter |
| ASV_607 | 12:00 | -2.75 | Decreasing | Proteobacteria | Alphaproteobacteria | Sphingomonadales | Sphingomonadaceae | Unclassified |
| ASV_1313 | 18:00 | -2.68 | Decreasing | Acidobacteriota | Acidobacteriae | Subgroup_2 | Subgroup_2 | Subgroup_2 |
| ASV_1799 | 12:00 | -2.68 | Decreasing | Acidobacteriota | Acidobacteriae | Bryobacterales | Bryobacteraceae | Bryobacter |
| ASV_611 | 12:00 | -2.66 | Decreasing | WPS-2 | WPS-2 | WPS-2 | WPS-2 | WPS-2 |
| ASV_1549 | 06:00 | -2.64 | Decreasing | Proteobacteria | Alphaproteobacteria | Caulobacterales | Caulobacteraceae | uncultured |
| ASV_1549 | 12:00 | -2.63 | Decreasing | Proteobacteria | Alphaproteobacteria | Caulobacterales | Caulobacteraceae | uncultured |
| ASV_1549 | 18:00 | -2.62 | Decreasing | Proteobacteria | Alphaproteobacteria | Caulobacterales | Caulobacteraceae | uncultured |
| ASV_2129 | 12:00 | -2.62 | Decreasing | Acidobacteriota | Acidobacteriae | Acidobacteriales | uncultured | uncultured |
| ASV_1337 | 06:00 | -2.62 | Decreasing | Proteobacteria | Alphaproteobacteria | Micropepsales | Micropepsaceae | uncultured |
| ASV_285 | 06:00 | -2.60 | Decreasing | Proteobacteria | Alphaproteobacteria | Caulobacterales | Caulobacteraceae | Caulobacter |
| ASV_1337 | 18:00 | -2.60 | Decreasing | Proteobacteria | Alphaproteobacteria | Micropepsales | Micropepsaceae | uncultured |
| ASV_2395 | 12:00 | -2.59 | Decreasing | Acidobacteriota | Acidobacteriae | Acidobacteriales | Acidobacteriaceae_(Subgroup_1) | Acidipila-Silvibacterium |
| ASV_2395 | 18:00 | -2.59 | Decreasing | Acidobacteriota | Acidobacteriae | Acidobacteriales | Acidobacteriaceae_(Subgroup_1) | Acidipila-Silvibacterium |
| ASV_309 | 06:00 | -2.58 | Decreasing | Proteobacteria | Gammaproteobacteria | Burkholderiales | Comamonadaceae | uncultured |
| ASV_994 | 18:00 | -2.58 | Decreasing | Proteobacteria | Alphaproteobacteria | Sphingomonadales | Sphingomonadaceae | Unclassified |
| ASV_607 | 18:00 | -2.57 | Decreasing | Proteobacteria | Alphaproteobacteria | Sphingomonadales | Sphingomonadaceae | Unclassified |
| ASV_3588 | 06:00 | -2.57 | Decreasing | Proteobacteria | Alphaproteobacteria | Micropepsales | Micropepsaceae | uncultured |
| ASV_2597 | 12:00 | 2.60 | Increasing | Proteobacteria | Gammaproteobacteria | Burkholderiales | Burkholderiaceae | Burkholderia-Caballeronia-Paraburkholderia |
| ASV_2394 | 12:00 | 2.73 | Increasing | Acidobacteriota | Acidobacteriae | Bryobacterales | Bryobacteraceae | Bryobacter |
| ASV_3199 | 12:00 | 2.76 | Increasing | Acidobacteriota | Acidobacteriae | Subgroup_2 | Subgroup_2 | Subgroup_2 |
| ASV_317 | 12:00 | 2.88 | Increasing | Actinobacteriota | Acidimicrobiia | uncultured | uncultured | uncultured |
| ASV_806 | 06:00 | 3.18 | Increasing | Proteobacteria | Alphaproteobacteria | Acetobacterales | Acetobacteraceae | Acidocella |
| ASV_319 | 12:00 | 3.22 | Increasing | Actinobacteriota | Thermoleophilia | Solirubrobacterales | Solirubrobacteraceae | Conexibacter |


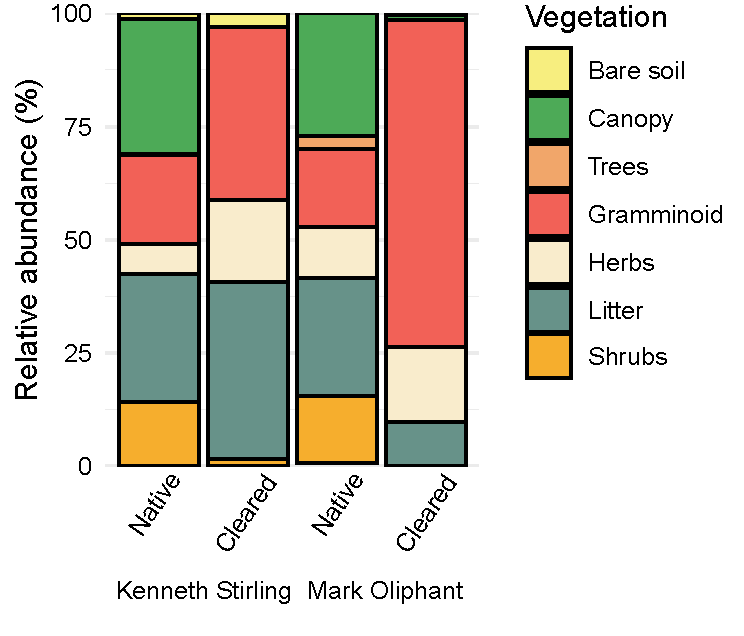


**Figure S1.** Relative abundance of vegetation growth forms at Kenneth Stirling CP and Mark Oliphant CP for cleared and native land use types.


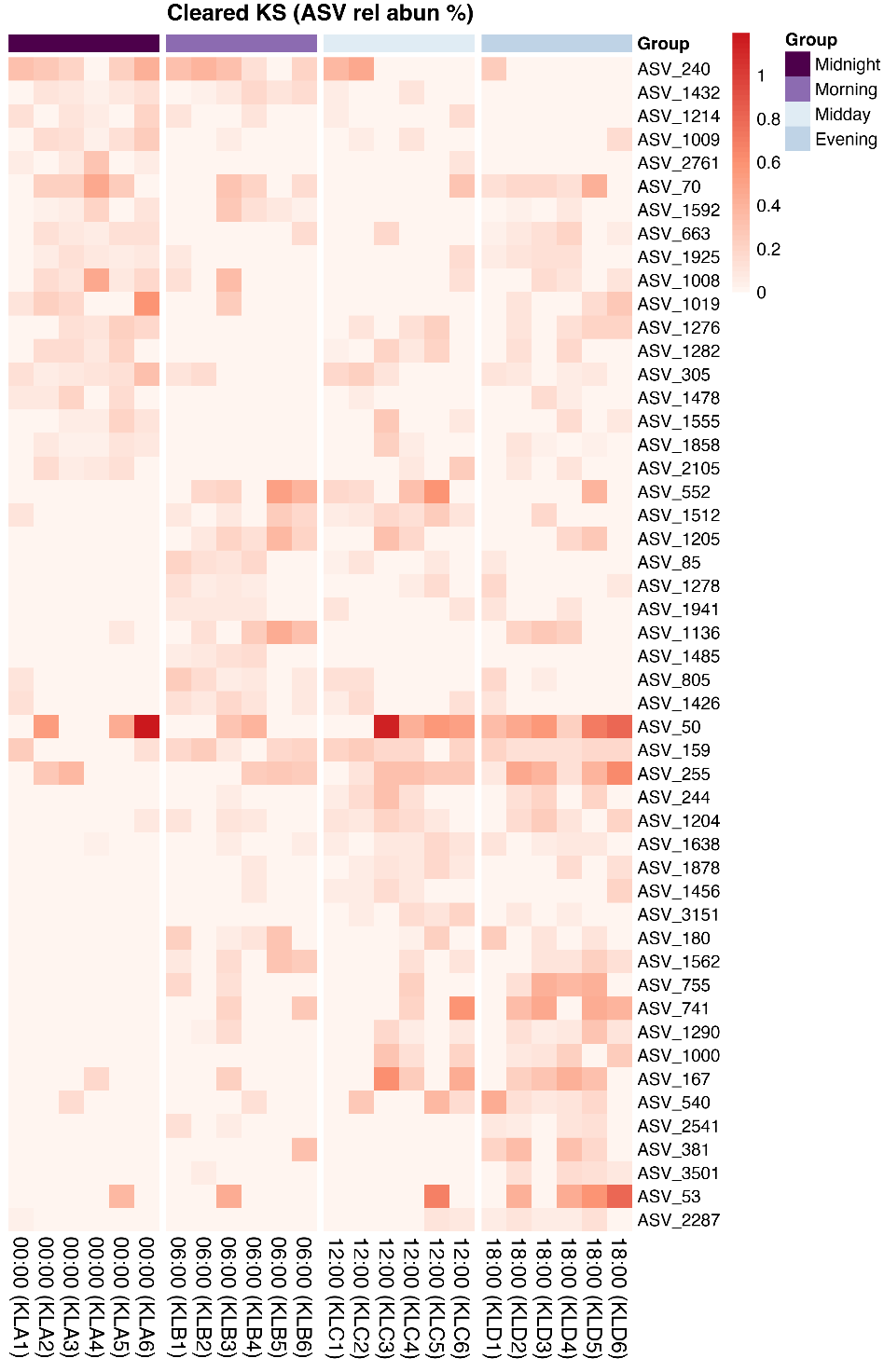

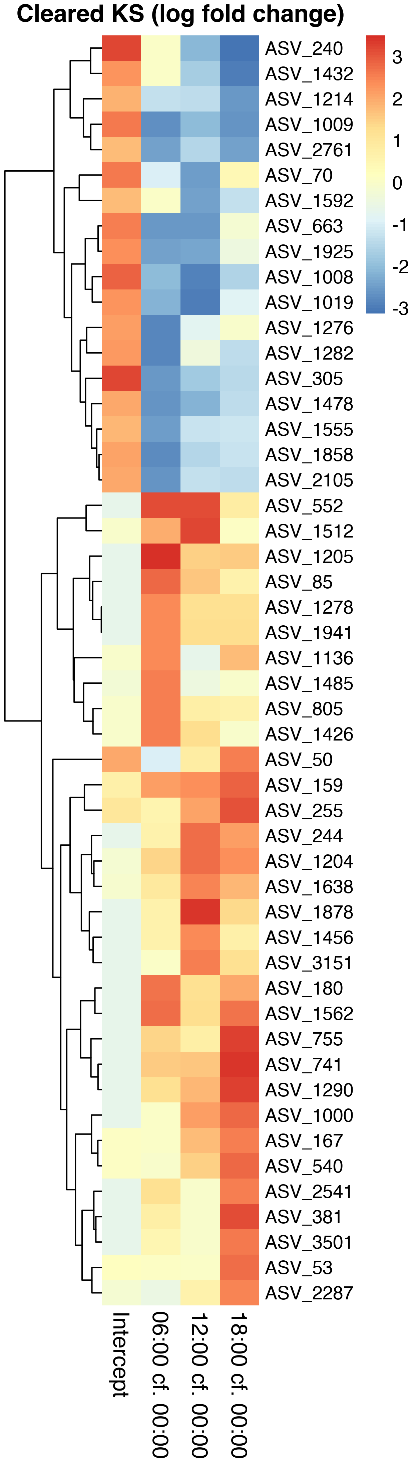


**Figure S2.** Cleared Kenneth Stirling sample (*n* = 6 for each sampling time) ASV relative abundances (%) and log-fold-change results from ANCOM-BC differential abundance testing for the 50 taxa displaying the largest magnitude of log-fold change between comparison groups. Intercept values reflect high or low abundance values in the baseline 00:00 hr group. Taxa (rows) are ordered by default log-fold-change heatmap clustering (right panel).0


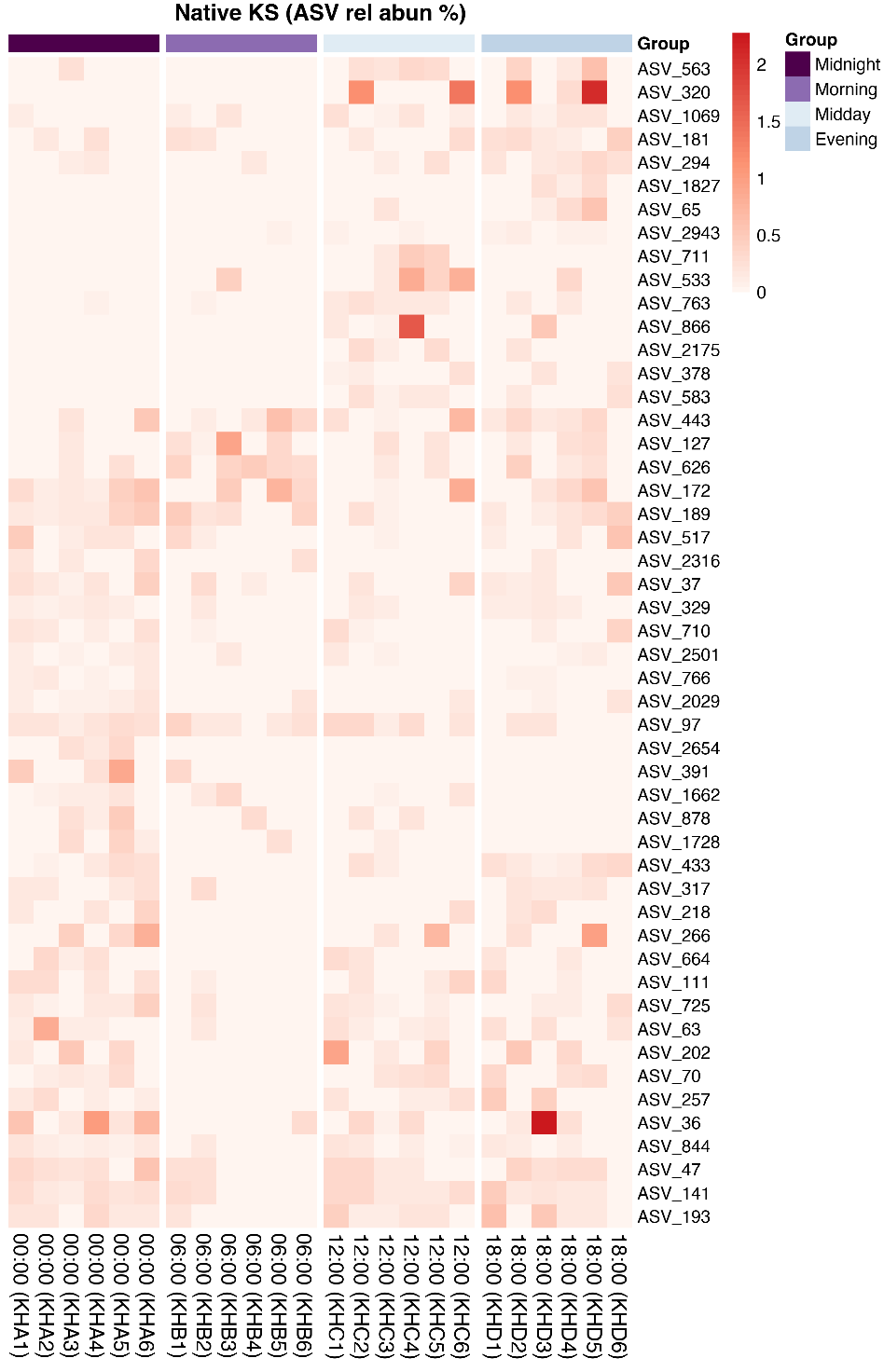

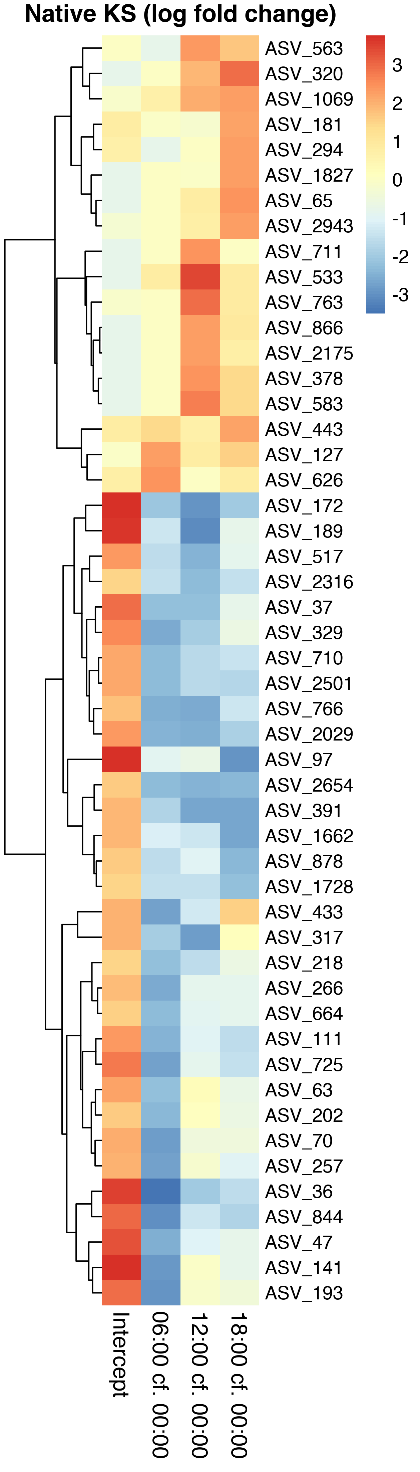


**Figure S3.** Native Kenneth Stirling sample (*n* = 6 for each sampling time) ASV relative abundances (%) and log-fold-change results from ANCOM-BC differential abundance testing for the 50 taxa displaying the largest magnitude of log-fold change between comparison groups. Intercept values reflect high or low abundance values in the baseline 00:00 hr group. Taxa (rows) are ordered by default log-fold-change heatmap clustering (right panel).


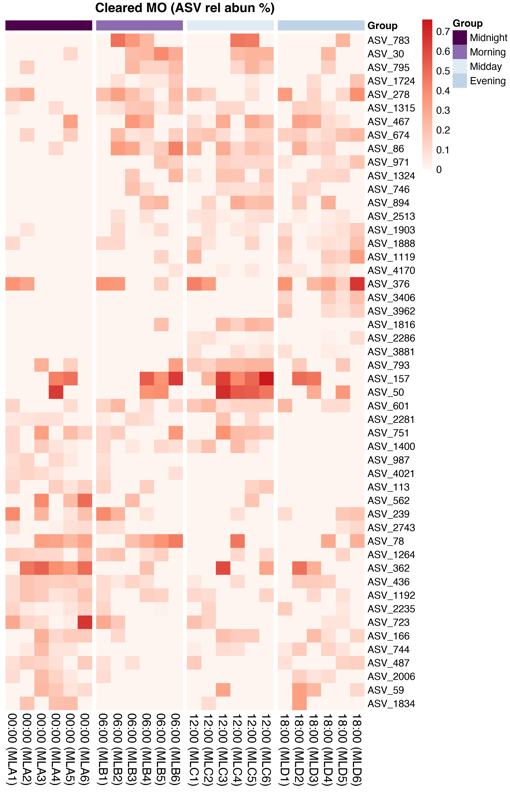

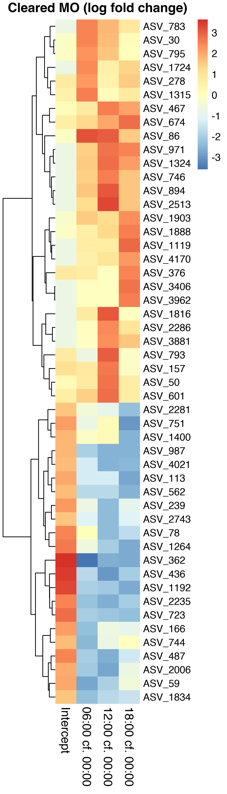


**Figure S4.** Cleared Mark Oliphant sample (*n* = 6 for each sampling time) ASV relative abundances (%) and log-fold-change results from ANCOM-BC differential abundance testing for the 50 taxa displaying the largest magnitude of log-fold change between comparison groups. Intercept values reflect high or low abundance values in the baseline 00:00 hr group. Taxa (rows) are ordered by default log-fold-change heatmap clustering (right panel).


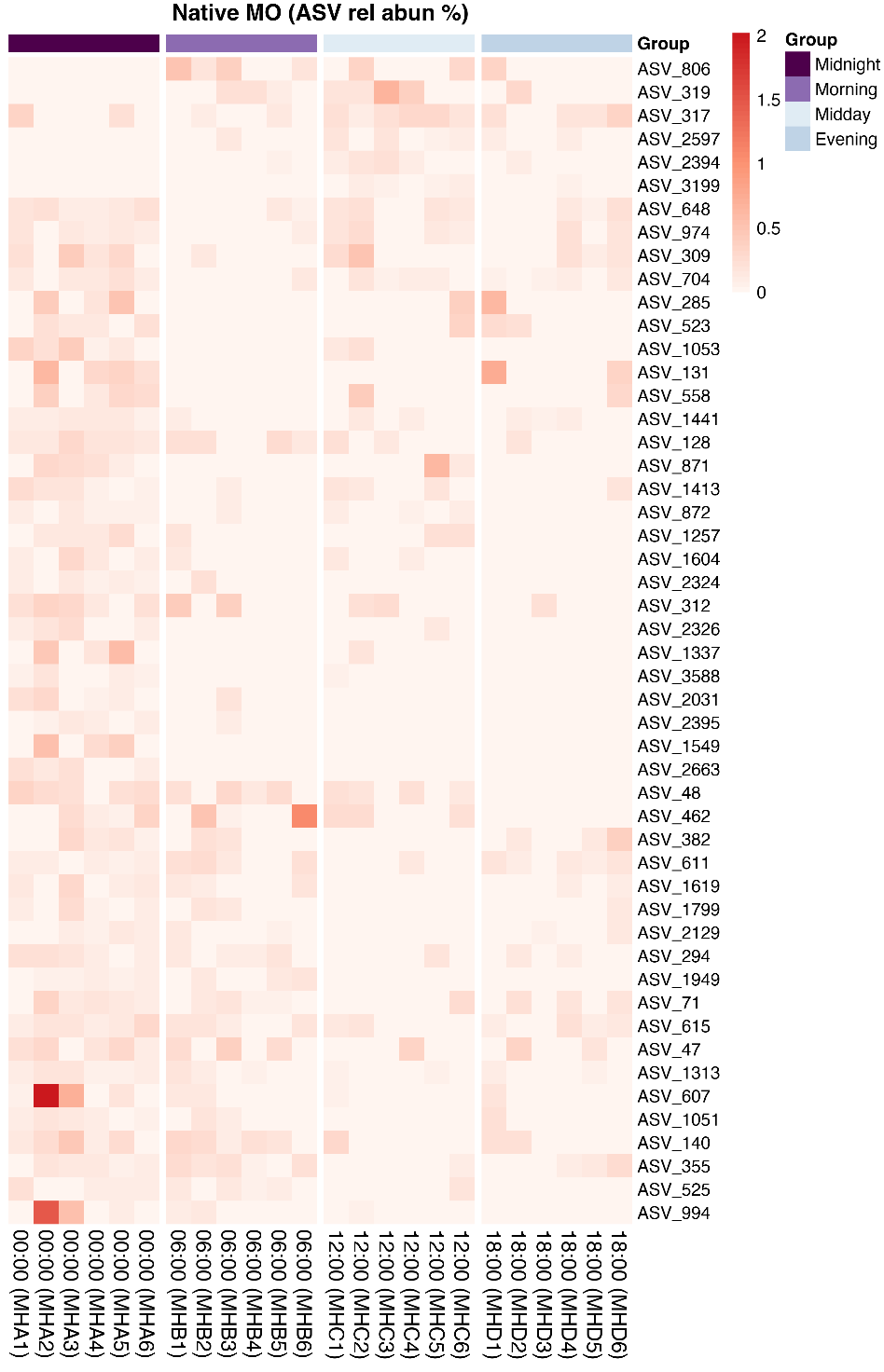

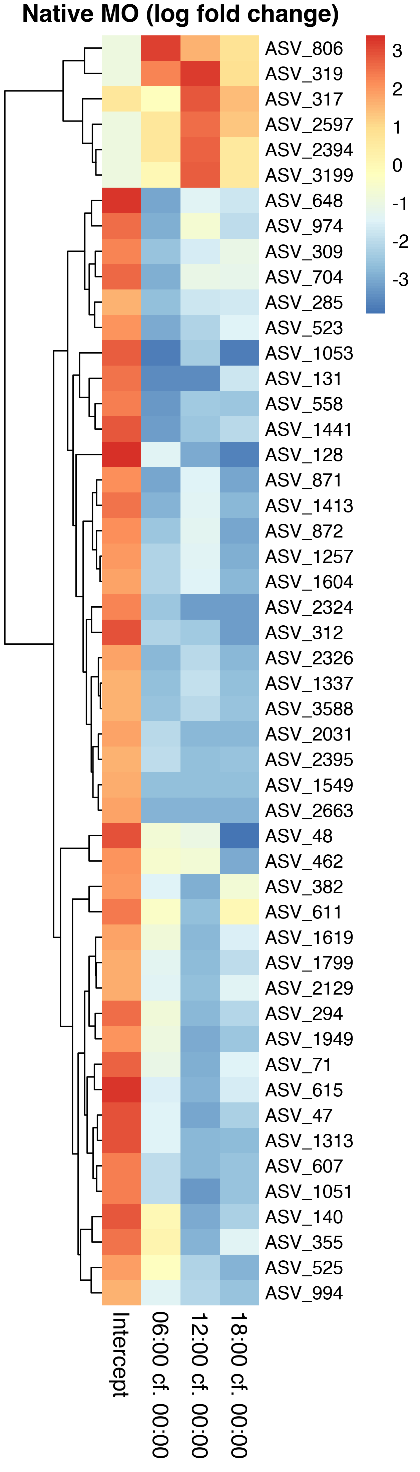


**Figure S5.** Native Mark Oliphant sample (*n* = 6 for each sampling time) ASV relative abundances (%) and log-fold-change results from ANCOM-BC differential abundance testing for the 50 taxa displaying the largest magnitude of log-fold change between comparison groups. Intercept values reflect high or low abundance values in the baseline 00:00 hr group. Taxa (rows) are ordered by default log-fold-change heatmap clustering (right panel).

|  | INCREASING  cf. 00:00 | DECREASING  cf. 00:00 |
| --- | --- | --- |
| @ 06:00 | 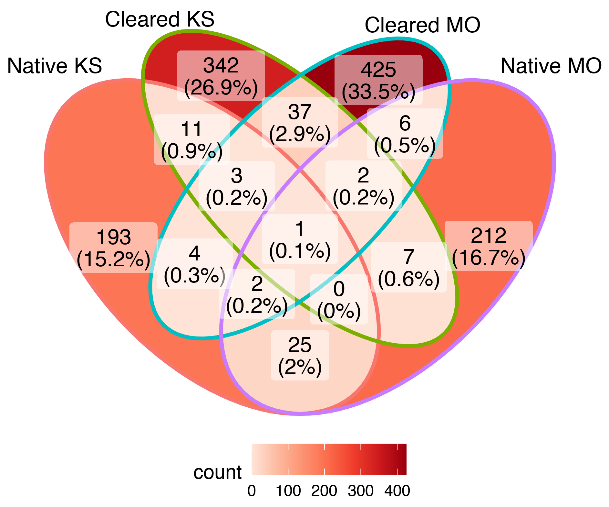 | 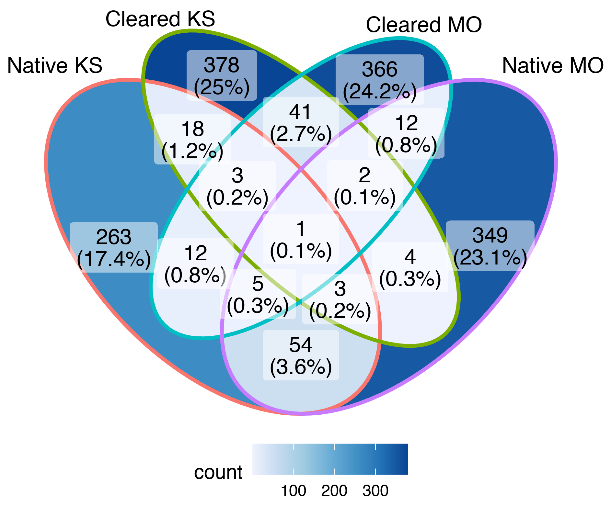 |
| @ 12:00 | 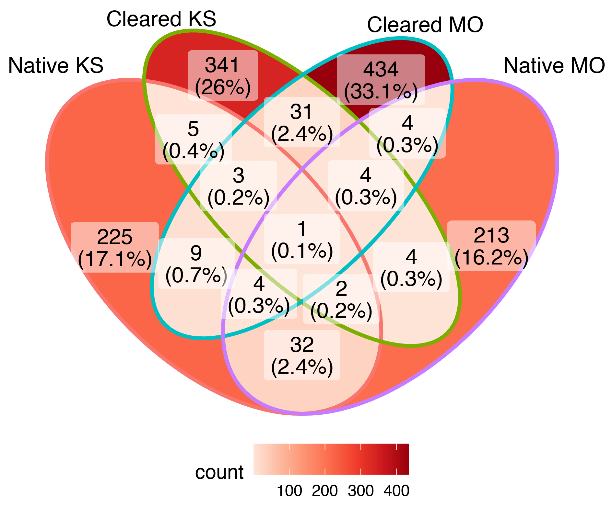 | 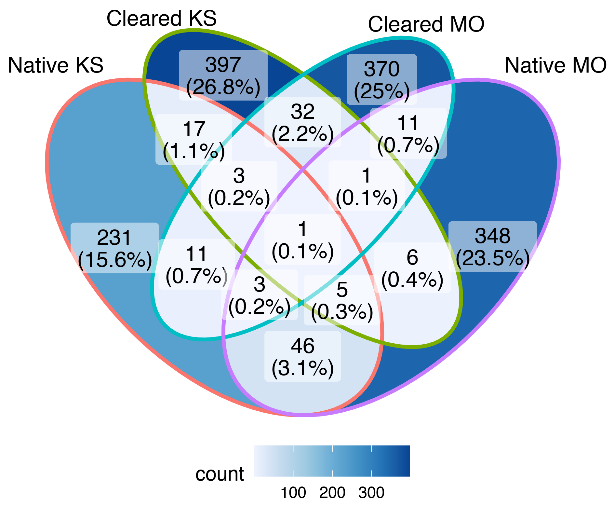 |
| @ 18:00 | 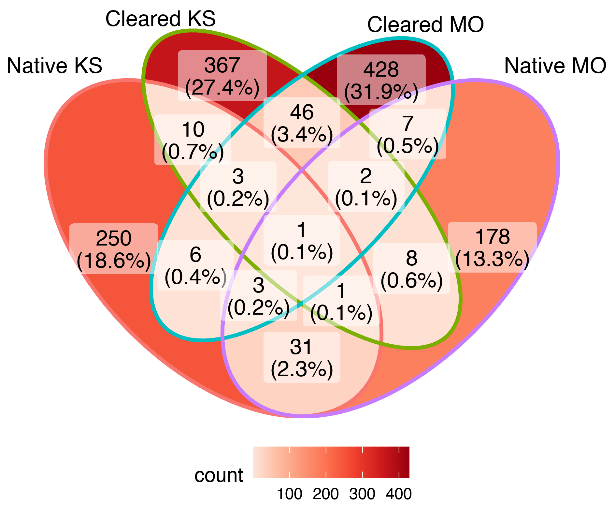 | 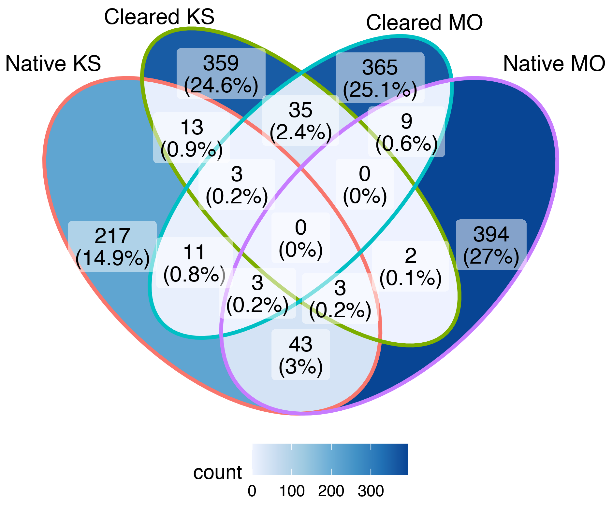 |

**Figure S6.** Venn diagrams displaying the number of overlapping ASVs across site types (Native KS, Cleared KS, Cleared MO, Native MO) that are identified as differentially abundant compared to the baseline sampling time at midnight (00:00) when measured at later sampling times (06:00, 12:00, 18:00).
